# Monitoring interfacial electric fields at a hematite electrode during water oxidation†

**DOI:** 10.1039/d2sc05628c

**Published:** 2023-02-23

**Authors:** Khezar H. Saeed, Dora-Alicia Garcia Osorio, Chao Li, Liam Banerji, Adrian M. Gardner, Alexander J. Cowan

**Affiliations:** a Department of Chemistry, Stephenson Institute for Renewable Energy, University of Liverpool Liverpool UK acowan@liverpool.ac.uk; b Early Career Laser Laboratory, University of Liverpool Liverpool UK

## Abstract

To understand the mechanisms of water oxidation on materials such as hematite it is important that accurate measurements and models of the interfacial fields at the semiconductor liquid junction are developed. Here we demonstrate how electric field induced second harmonic generation (EFISHG) spectroscopy can be used to monitor the electric field across the space-charge and Helmholtz layers in a hematite electrode during water oxidation. We are able to identify the occurrence of Fermi level pinning at specific applied potentials which lead to a change in the Helmholtz potential. Through combined electrochemical and optical measurements we correlate these to the presence of surface trap states and the accumulation of holes (h^+^) during electrocatalysis. Despite the change in Helmholtz potential as h^+^ accumulate we find that a population model can be used to fit the electrocatalytic water oxidation kinetics with a transition between a first and third order regime with respect to hole concentration. Within these two regimes there are no changes in the rate constants for water oxidation, indicating that the rate determining step under these conditions does not involve electron/ion transfer, in-line with it being O–O bond formation.

## Abstract 2

Electric field induced second harmonic generation spectroscopy is used to monitor the potential drop across the space-charge and Helmholtz layers in a hematite electrode during water oxidation.

## Graphical abstract

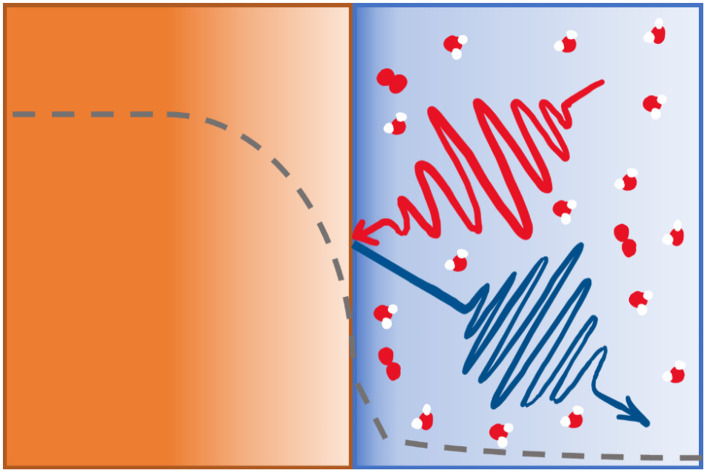

## Introduction

Hematite (α-Fe_2_O_3_) is a promising material for water oxidation. The low cost, good stability and high abundance of hematite, coupled to its suitable band gap for visible light absorption has led to massive efforts towards engineering more efficient photoelectrodes.^1^ Mechanistic studies of hematite electrodes, and photoelectrodes aim to elucidate the underlying physico-chemical phenomena controlling water splitting efficiencies which is vital in enabling the design of the next generation of photoelectrodes.

**Fig. 1:**
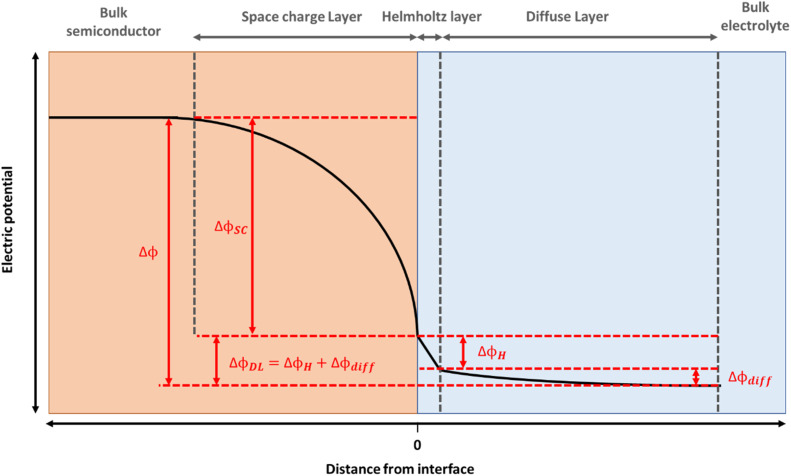
Potential distribution at a typical n-type semiconductor|electrolyte junction, where Δ*ϕ*_SC_ represents the potential drop over the space charge layer, Δ*ϕ*_H_ over the Helmholtz layer and Δ*ϕ*_diff_ over the diffuse layer (such that the potential drop over the double layer, Δ*ϕ*_DL_ = Δ*ϕ*_H_ + Δ*ϕ*_diff_). The high electrolyte concentrations employed allow us to approximate Δ*ϕ* ∼ Δ*ϕ*_H_ + Δ*ϕ*_SC_.

The electrocatalytic and photoelectrochemical properties of a semiconductor are profoundly affected by the presence of the interfacial electric fields at the semiconductor liquid junction (SCLJ). For hematite, an n-type semiconductor, a depletion of charge carriers within its structure occurs when contacted to an aqueous electrolyte and a positive space charge layer is formed between the bulk of the semiconductor and the electrolyte interface.^2^ This positive charge is balanced by a compact layer of negatively charged ions in the electrolyte (the Helmholtz or Stern layer) and a diffuse layer of charges towards the bulk of the electrolyte, with the resulting potential distribution shown in Fig. 1. For concentrated electrolytes, such as those used here, the potential drop within the electrolyte is primarily across the Helmholtz layer (Δ*ϕ*_H_). Thus, for these electrodes there is a distribution in the electric field along the surface normal direction and understanding how this varies as illumination and/or the potential applied to the electrode is changed is essential if we are to rationalise how a (photo)electrode behaves. But it is challenging to directly monitor these fields under operating (water oxidation) conditions.

Capacitance measurements such as Mott–Schottky analyses can be used to study hematite electrodes but they typically require an assumption of the potential distribution across the Helmholtz and space-charge layers. Mott–Schottky analyses are also complicated by the presence of surface and defect states and it has been shown that for hematite, a material where surface states play a key role in determining photoelectrochemical performance, these measurements can give unreliable results.^3^ Transient absorption spectroscopy with a UV/vis probe has been used extensively to understand how photogenerated charge carrier dynamics change as the applied potential is altered.^4–7^ But transient absorption spectroscopy does not directly probe the different fields present. These are instead inferred from the applied potential dependence of the kinetics of the charge carriers. Here we use electric field-induced second harmonic generation (EFISHG) spectroscopy to study a hematite electrode to report on the changes in potential across the Helmholtz and space-charge layers under operating conditions as hole (h^+^) accumulation occurs at the SCLJ.

Understanding how h^+^ accumulation changes the potential distribution across the SCLJ and the subsequent impact of this on water oxidation kinetics is important. It is widely accepted that the slow kinetics of water oxidation, with h^+^ lifetimes at the SCLJ of milliseconds reported,^8–11^ limits the efficiency of metal oxide photoanodes. With conventional metallic electrodes the kinetics of electrocatalytic reactions are often interpreted using the Butler–Volmer equation. In this case application of a potential leads to a change in Δ*ϕ*_H_ causing a change in the energetics (and hence rate constant) for charge transfer giving rise to an exponential increase in current with applied potential. In contrast for an idealized semiconductor (*e.g.* no surface states present, low doping concentration) in a concentrated electrolyte the majority of the potential drop across the SCLJ is expected to occur across the space charge region with minimal change to Δ*ϕ*_H_. The invariance of Δ*ϕ*_H_ means that the rate constant for electron transfer can be considered independent of applied potential. Instead of using the Butler–Volmer model well established population models can be used,^12,13^ where the change in rate of reaction is due to a change in surface electron/hole population. Recently Durrant *et al.* used a population model (eqn (1)) to correlate optically measured [h^+^] to rates of water oxidation (*J*^wo^) for a range of metal oxide electrodes and photoelectrodes including hematite.^11,14,15^ These experiments indicated that a switch in mechanism occurs on hematite photoelectrodes with the reaction order (*α*) with respect to [h^+^] being ∼3 at high [h^+^] (occurring at ∼1 sun), but ∼1 at low [h^+^] and that the water oxidation rate constant (*k*_wo_) for each pathway is independent of applied potential.^15^ This behaviour has been interpreted to indicate a change in water oxidation mechanism from occurring at a single Fe centre to a mechanism involving interaction between two adjacent Fe^IV^=O sites (the proposed surface trapped h^+^) when the surface coverage of h^+^ reaches a high enough level. Further studies using transient photocurrent measurements have since also reported a third-order dependence of *J*^wo^ on hole density with DFT calculations showing O–O bond formation occurring at sites where three oxo/oxyl groups are found.^16^

1 *J*^wo^ = *k*_wo_ × [h^+^]^*α*^

Supporting the use of a population model are measurements of hole activation energies which are constant at a range of applied potentials with α-Fe_2_O_3_,^17^ which could indicate that minimal change in Δ*ϕ*_H_ occurs as [h^+^] changes. However, as recently highlighted by Zhang and Leng,^18^ it is surprising that a population model holds for hematite and other oxide photoelectrodes. When the rate of h^+^ transfer is slow, h^+^ accumulation causes light-induced Fermi level pinning. Fermi level pinning leads to changes in Δ*ϕ*_H_ as the applied potential is changed, which in turn might be expected to change *k*_wo_. Supporting the concerns of Zhang and Leng is that in, several studies fitting of Intensity-Modulated Photocurrent Spectroscopy (IMPS) and Photoelectrochemical Impedance Spectroscopy (PEIS) data of hematite electrodes gives *k*_wo_ values that are dependent on applied potential.^19–23^ It is therefore clear that to validate the proposed models of electron transfer at the hematite–water interface new measurements that directly address the local electric fields whilst monitoring the h^+^ population are required.

Second harmonic generation (SHG) is a second order nonlinear optical process where the electric field of incident light at frequency *ω*_i_ induces a polarisation within the material and light is emitted at double this frequency (*ω*_SHG_ = *ω*_2i_).^24^ The intensity of the SHG (*I*_SHG_) signal is proportional to the square of the second order polarisation of the material (*P*^(2)^), which in turn is dependent on both the electric field of the incoming photons and the second order susceptibility (*χ*^(2)^) of the system under study:

2 *I*_SHG_ ∝ [*P*^(2)^]^2^ = [*χ*^(2)^***E***(*ω*_i_)***E***(*ω*_i_)]^2^

Due to inversion symmetry, *χ*^(2)^ = 0 in centrosymmetric materials and only becomes non-zero at interfaces, where the inversion symmetry is inherently broken. SHG spectroscopy has been used previously to study a variety of catalytic systems at interfaces including monitoring adsorption of gaseous reactants at solid surfaces and even identifying reaction intermediates in liquid phases (when the intermediates have a resonance at the second harmonic wavelength).^25,26^ This surface-selectivity makes SHG spectroscopy especially useful for studying electrochemical systems. EFISHG is a form of SHG in which the response of a charged interface requires consideration of an additional static electric field(s) (***E***_DC_), eqn (3). The presence of additional fields can influence *I*_SHG_ through two mechanisms.^27^ Firstly, by directly interacting with bulk third order susceptibility (*χ*^(3)^) which does not usually contribute to the SHG response, but is induced by the additional static field. Thus, the presence of ***E***_DC_ means non-interfacial species will contribute to the second harmonic response, resulting in a change in the SHG intensity. Secondly, the presence of ***E***_DC_ can also change the net orientation of mobile interfacial molecular species, or for a material such as a semiconductor electrode induces a polarization of the lattice, manifesting as a change in *χ*^(2)^. Thus, the second harmonic polarisation for an electrode held at a potential (*ϕ*) can be summarised as:

3 *P*_SHG_ = *χ*^(2)^(*ϕ*)***E***(*ω*)***E***(*ω*) + *χ*^(3)^(*ϕ*)***E***(*ω*)***E***(*ω*)***E***_DC_

The dependence of the second harmonic polarisation on the interfacial electric field has been used extensively to study the evolution of electric fields at metal electrode|electrolyte interfaces based on the nonlinear electroreflectance studies by Bloembergen *et al.*^28,29^ However, fewer studies have exploited EFISHG to study the semiconductor electrode|electrolyte interface.^30–33^ Experiments on a TiO_2_ electrode^34^ reported a linear relationship between the applied potential and *I*_SHG_ at potentials positive of flat band (*ϕ*_fb_). To rationalise this behaviour, it is important to first consider the relationship between applied potential (Δ*ϕ*, relative to the reference electrode) and the strength of the electric field across the space charge layer. The Mott–Schottky approximation tells us that for a n-type semiconductor that is held positive of the flat band potential (*ϕ*_fb_), the electric field varies linearly over the space charge layer and the charge (*q*_SC_) is proportional to the square root of Δ*ϕ*_SC_ (where Δ*ϕ*_SC_ = *ϕ* − *ϕ*_fb_), eqn (4).

4 *q*_SC_ = (2*εε*_0_*eN*_D_)^1/2^(*ϕ* − *ϕ*_fb_)^1/2^

In this case the overall charge of the space charge layer is directly related to the magnitude of the electric field (eqn (5)),^30,34^ and it is possible to arrive at eqn (6) from eqn (3), assuming that the potential drop is solely over the space charge layer:5
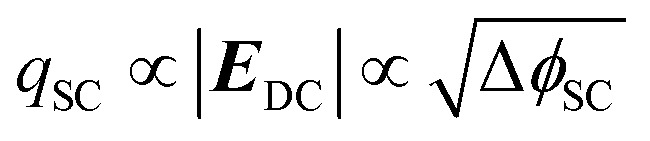
6
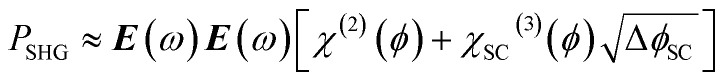


As *I*_SHG_ is proportional to the square of *P*_SHG_, eqn (6) has been used to conclude that for the TiO_2_ electrode studied previously the potential drop was primarily across the space charge layer at potentials positive of flat band and that the *χ*^(3)^ contribution was dominant in the EFISHG response.^34^ A similar linear dependence of *I*_SHG_ with applied potential for a n-Si positive of flat-band has also been reported.^35^ Interestingly, at potentials negative of flat-band a non-linear (parabolic) potential dependence of *I*_SHG_ occurred, which was rationalised by the electrode becoming more metal like under these conditions leading to Δ*ϕ*_H_ changing with applied potential instead of Δ*ϕ*_SC_.^28,30^ These past studies demonstrate that EFISHG can be used to study the SCLJ and to monitor the distribution of the potential drop, but to the best of our knowledge EFISHG has not been used to study electrodes during water oxidation. SHG has also been used to study the interactions of dissolved ions with hematite.^36,37^ Here we combine SHG spectroscopy with optical/kinetic measurements of [h^+^] to follow how the potential drop across the SCLJ varies for a hematite electrode as h^+^ accumulate at the SCLJ. We show that h^+^ accumulation leads to changes in Δ*ϕ*_H_ but that the water oxidation rate constant is independent of Δ*ϕ*_H_ when there is a first or third-order dependence of *J*^wo^ on [h^+^], indicating that under these conditions the rate determining step does not involve electron/ion transfer, in-line with it being O–O bond formation.

## Results & discussion

### EFISHG response of hematite

**Fig. 2:**
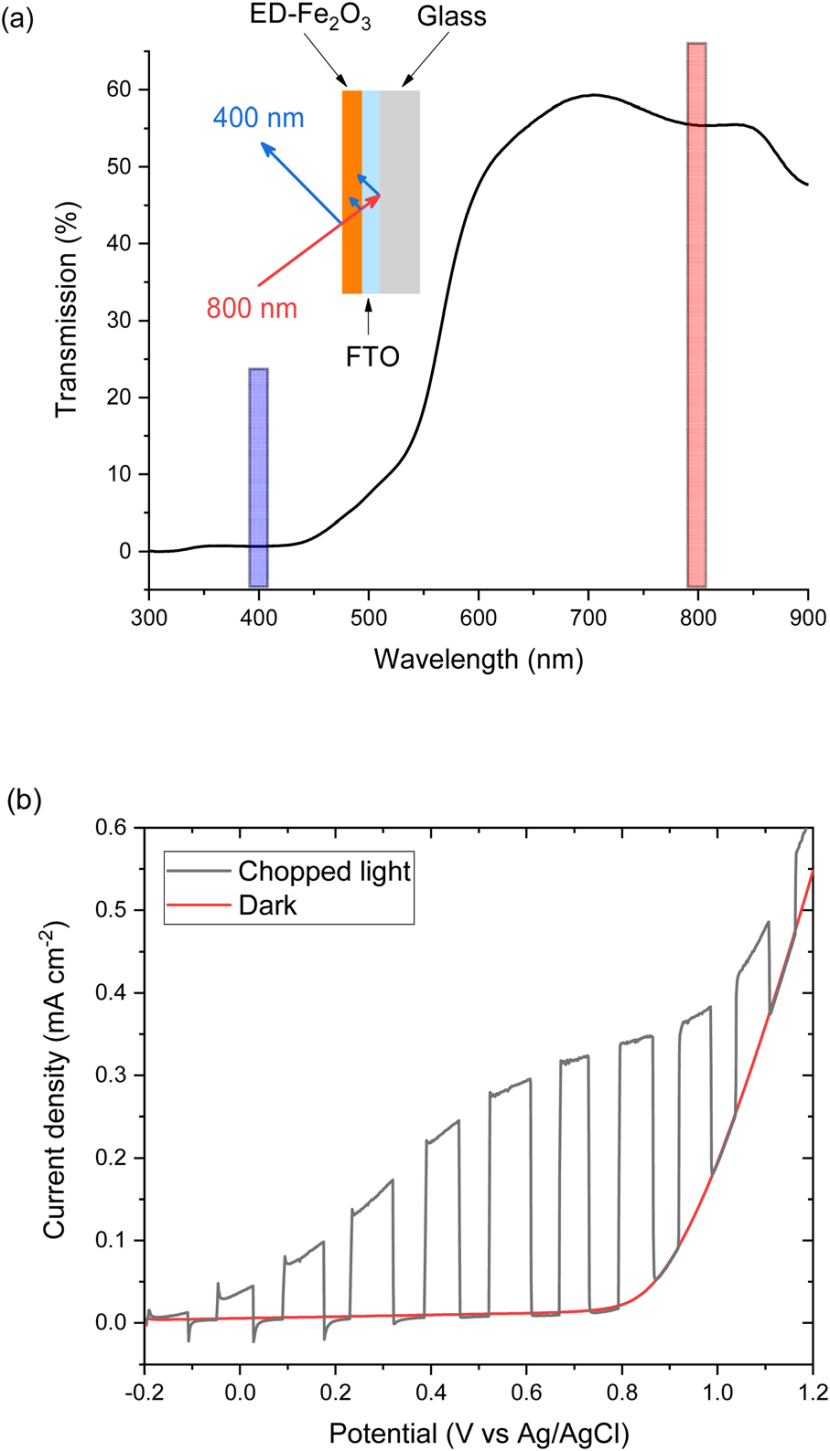
(a) UV-vis in transmission of the α-Fe_2_O_3_ electrode used for SHG experiments. Inset, experimental geometry for reflection SHG experiments, highlighting the absorption of SHG photons (blue) from the buried interface by the layer of hematite on commercial FTO-coated glass. (b) Chopped light (15 mW cm^−2^, 365 nm) and dark linear sweep voltammograms (LSVs) at 10 mV s^−1^ of α-Fe_2_O_3_ electrode in 0.1 M NaOH.

EFISHG experiments (see Fig. S1 and S2† for more details on the SHG experimental setup) were carried out on electrodeposited α-Fe_2_O_3_ electrodes on FTO (FTO = F-doped SnO_2_) glass. Full details of the synthesis and characterisation of the photoelectrodes are found in the Experimental section and ESI (Fig. S3–S10).† The samples consist of α-Fe_2_O_3_ layers (150 nm thick) that have low surface roughness (Fig. S3 and S5†) making them suitable for the EFISHG measurements and they also exhibit a reasonable level of photoelectrochemical activity (photocurrent onset ∼ −0.2 V_Ag/AgCl_ ∼ 0.8 V_RHE_, 0.33 mA cm^−2^ under 15 mW cm^−2^ 365 nm illumination, Fig. 2b). As is typically the case for a (photo)electrode these samples contain multiple SHG-active interfaces (electrolyte|α-Fe_2_O_3_|FTO|glass) in close proximity, Fig. 2a. Careful selection of the incident radiation wavelength can allow only the interface of interest to be probed. With a sufficiently thick layer of α-Fe_2_O_3_, the electrode itself can act as an optical filter for the (unwanted) underlying interfaces. An incident wavelength of 800 nm was selected for these experiments, generating SHG photons at 400 nm. The absorption coefficient for α-Fe_2_O_3_ is reported to be *α* ∼ 2 × 10^5^ cm^−1^ (*α*^−1^ ∼ 29 nm) at 400 nm.^38^ Therefore, any SHG from the α-Fe_2_O_3_|FTO and FTO|glass interfaces is almost completely absorbed by the hematite layer in these electrodes and it does not contribute to the detected EFISHG response. Photoelectrochemical tests indicate that when the 800 nm laser (2.5 μJ per pulse, 170 fs pulse duration, 10 kHz) is incident on the α-Fe_2_O_3_ there is no detectable photocurrent (>30 nA, Fig. S8†) indicating that neither the 800 nm or 400 nm SHG generated is able to lead to a significant change in the steady-state carrier population. The incident and SHG wavelengths are also not on resonance with trapped h^+^ which have a maximum in absorbance at *ca.* 625 nm.^8,15^

**Fig. 3:**
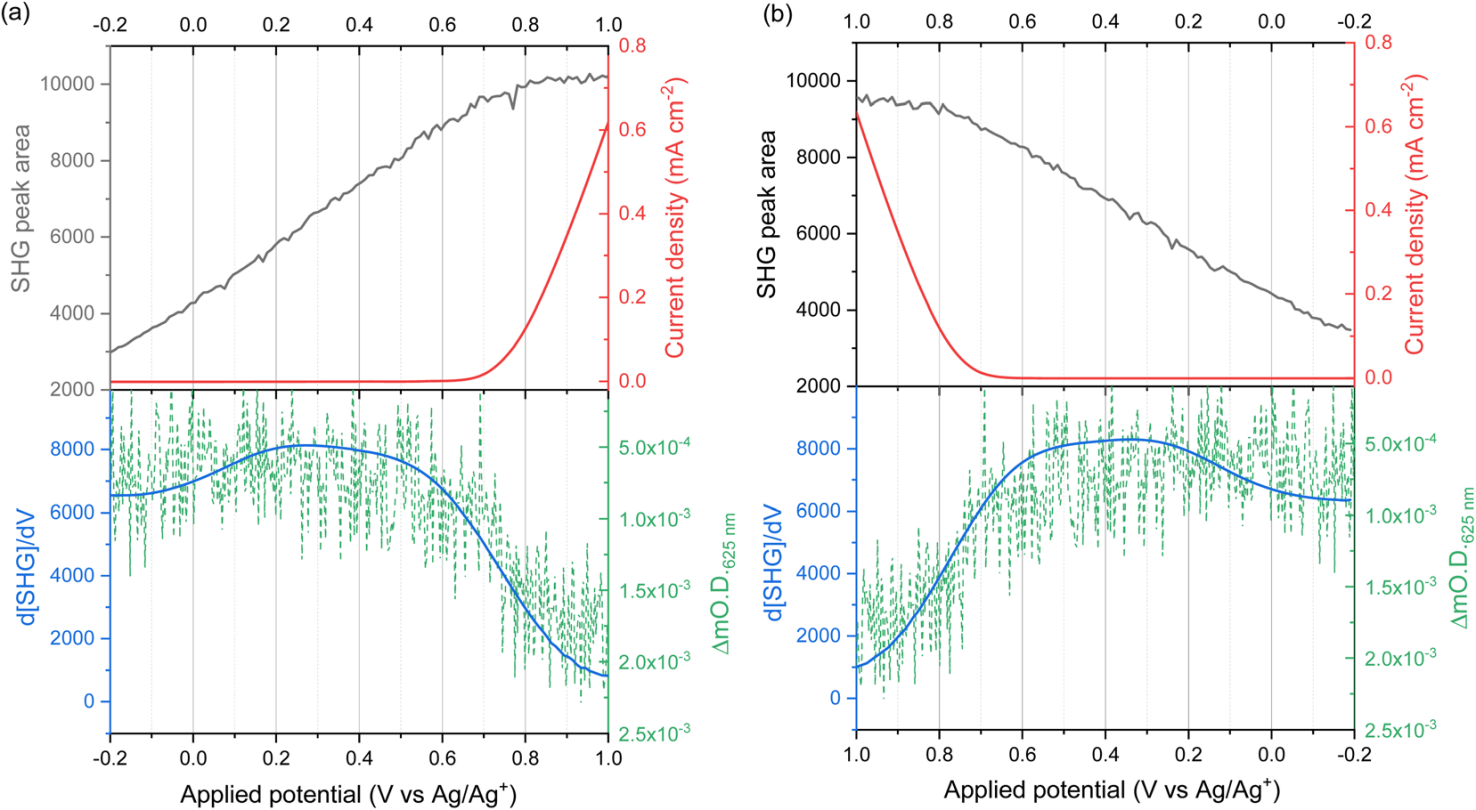
*I*_SHG_ (grey line) recorded at 400 nm (2.5 μJ per pulse p-polarised 800 nm incident light at 10 kHz) during cyclic voltammogram at 2 mV s^−1^ in 0.1 M NaOH. (a) Shows the data recorded in the outward (−0.2 to 1.0 V *vs.* a Ag/Ag^+^ pseudo reference electrode) sweep, (b) shows the data for the return (1.0 to −0.2 V) sweep, demonstrating the reversibility of the SHG response. The current density is shown by the red line. The lower panels show the rate of change of *I*_SHG_ (blue line) and the change in optical density at 625 nm (green dashed line) recorded during a subsequent linear sweep voltammogram at 2 mV s^−1^ in 0.1 M NaOH. The change in optical density at 625 nm (compared to the same electrode at open circuit potential) is a probe of hole density. Note, the green (mΔOD) axes are reversed to better display the correlation of an increase in hole concentration with a decrease in d[SHG]/d*V*.

The potential dependence of the SHG response of the α-Fe_2_O_3_ electrode in the dark between −0.2 and 1.0 V_Ag/Ag^+^_ is shown in Fig. 3a and b. The limitations of the SHG cell mean that a Ag pseudo reference electrode is used but as discussed alongside Fig. S9† the potential of the Ag/Ag^+^ ∼ −0.1 V Ag/AgCl in this experiment. The flat band potential of this electrode is found to be *ca.* −0.46 V_Ag/AgCl_ (Fig. S10†), therefore in the applied potential study (Fig. 3a and b) the α-Fe_2_O_3_ electrode is always positive of flat band. In Fig. 3a and b we see a potential dependence of *I*_SHG_ from the electrolyte|α-Fe_2_O_3_|FTO|glass sample. Comparison of the potential dependent SHG response to an electrolyte|FTO|glass electrode (Fig. S11b†) shows clear differences, indicating that we are able to successfully monitor the SHG response of the α-Fe_2_O_3_|electrolyte interface. The lower panels of Fig. 3a and b show the rate of change of *I*_SHG_ from the α-Fe_2_O_3_|electrolyte interface with applied potential. *I*_SHG_ changes at a roughly constant, linear rate with applied potential between 0.2 and 0.6 V_Ag/Ag^+^_ and also between −0.2 and −0.05 V_Ag/Ag^+^_. As outlined in the introduction a linear response in *I*_SHG_ with applied potential has been proposed to be the result, and an indicator, of the potential drop occurring across the space charge region and of the *χ*^(3)^ contribution dominating the EFISHG response (eqn (6)).^34^ Here we also conclude that between 0.2 to 0.6 V_Ag/Ag^+^_ and −0.2 to −0.05 V_Ag/Ag^+^_ the potential drop is primarily across the space charge region and that the *χ*^(3)^ term arising from the space charge layer is leading to the potential dependent *I*_SHG_. A full expansion of eqn (6) and discussion of the relative contributions of the 2^nd^ and 3^rd^ order terms can be found in the ESI.†

**Fig. 4:**
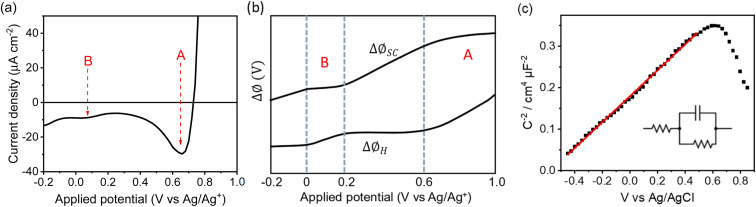
(a) Fast (200 mV s^−1^) reverse LSV of an α-Fe_2_O_3_ electrode in 0.1 M NaOH from 1.2 V to −0.2 V *vs.* Ag/Ag^+^ after holding at 1.2 V for 1 minute to build up a large population of trapped holes (region (A)). The measurement also highlights a surface electron trap state (B). (b) Qualitative distribution of potential across the space charge and Helmholtz layer derived from the SHG data. In regions A and B Fermi level pinning occurs leading to the potential drop occurring over the Helmholtz layer. (c) Mott–Schottky analysis (using the circuit diagram in the inset) of the same hematite electrode in 0.1 M NaOH with a Ag/AgCl electrode, where the red line indicates a linear fit to the Mott–Schottky relation.

The deviation from a linear rate of change of *I*_SHG_ with applied electrode potential at >0.6 V_Ag/Ag^+^_ and between −0.05 and 0.2 V_Ag/Ag^+^_ may indicate an abrupt change where the majority of potential drop is occurring (*i.e. ϕ*Δ_H_*vs. ϕ*Δ_SC_). Supporting the possibility that a change in potential drop distribution is the Mott–Schottky analysis (Fig. 4c and S9†), which shows that at the more positive potentials examined there is a deviation from linearity. Between *ca.* 0.6 to 0.7 V_Ag/Ag^+^_ we see the onset of a catalytic current assigned to water electrolysis on the α-Fe_2_O_3_ surface. Dark water oxidation occurs at significant rates when the Fermi level approaches the valence band edge or a high enough density of suitably energetic inter-band states. At these very positive potentials the density of surface holes is expected to increase due to the slow rate of hole transfer during water oxidation on hematite.^15^ To monitor the [h^+^] we have measured the change in optical density at 625 nm during a linear sweep voltammogram. Here we find there is excellent agreement between the measured [h^+^] and the rate of change in *I*_SHG_ at potentials between ∼0.6 V to ∼1.0 V_Ag/Ag^+^_ in Fig. 3a. It is known that high levels of hole accumulation leads to Fermi-level pinning, with the effect being particularly pronounced when the electron transfer kinetics are slow,^39^ as is the case with α-Fe_2_O_3_.^8–10,15^ The h^+^ population begins to plateau at the highest potentials studied in Fig. 3 (1.0 V_Ag/Ag^+^_). Electrochemical measurements (see below, Fig. 4) demonstrate that between 0.6 and 1.0 V_Ag/Ag^+^_ the h^+^ are present in surface states and measurements of the optical density at 625 nm at higher potentials (Fig. S14†) 1.0–1.2 V_Ag/Ag^+^_ show that the valence band edge lies just positive of the region of study in the EFISHG experiment. Therefore, we conclude that the non-linear *I*_SHG_ response with applied potential at >0.6 V_Ag/Ag^+^_ is primarily due to the build-up of surface h^+^, changing the potential distribution across the interface, with the further changes in potential drop occurring across the Helmholtz layer instead of solely across the space-charge layer.

To identify if the deviation from the linear rate of change of *I*_SHG_ with applied potential between −0.05 to 0.2 V_Ag/Ag^+^_ is also due to Fermi level pinning, we have investigated the distribution of trap states on the hematite electrode. Using a methodology developed by Hamann *et al.,*^23^ we initially hold the electrode in the dark at a positive potential, where an OER current occurs, then the potential is rapidly (200 mV s^−1^) swept to more negative potentials depopulating (reducing) any oxidised trap states, Fig. 4a. Two different reductions can be seen in Fig. 4a. The feature at *ca.* 0.65 V_Ag/Ag^+^_ was previously assigned to the reduction of h^+^ that have accumulated at surface trap states,^23^ supporting our assignment of Femi level pinning and a change in *ϕ*Δ_H_ at >0.6 V_Ag/Ag^+^_ as a result of h^+^ accumulation. The 2^nd^ broad reduction centred around 0.1 V_Ag/Ag^+^_ in Fig. 4a has also been reported previously and assigned to surface electron/hole trap states.^23^ The good agreement between the potential of these electron/hole trap states and the deviation from the linear rate of change of *I*_SHG_ leads us to conclude that the lower rate of change of *I*_SHG_ potential between −0.05 to 0.2 V_Ag/Ag^+^_ is also due to Fermi-level pinning with the potential drop occurring across the Helmholtz layer, instead of solely over the space-charge layer.

### Analysis of surface mechanisms in the dark and under illumination

**Fig. 5:**
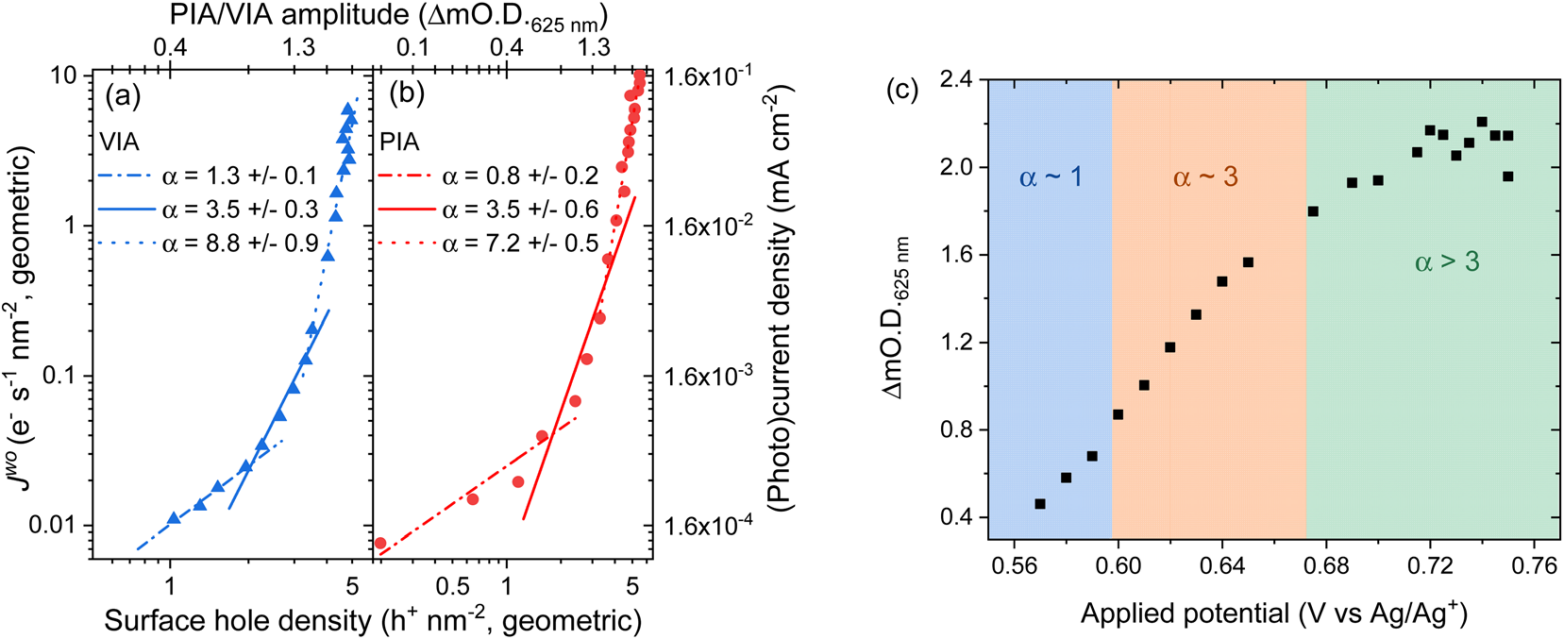
Log–log plot of current *versus* change in optical density at 625 nm for a hematite electrode in (a) the dark where the applied potential changes the water oxidation current and hole density (voltage induced absorption, VIA data, blue) and (b) in the light where the electrode is held at 0.2 V_Ag/Ag^+^_ (positive of the onset of dark water oxidation) with the light (365 nm LED) intensity being modified between <1 to ∼30 mW cm^−2^ to change the photocurrent and hole density (PIA, red). The VIA data in the form of applied potential and change in optical density is shown in part (c) with the regions fitted to the different *α* values indicated by the shaded areas. Raw PIA/VIA data is shown in Fig. S16† and shown as a function of light intensity or voltage in Fig. S17.†

To understand the impact of the change in potential distribution on the mechanisms and kinetics of water oxidation we have carried out an operando spectroelectrochemistry study of the rate law, and examined its potential dependence, eqn (1). In the voltage induced absorption (VIA) experiment we correlated the steady-state current density (*J*^wo^) to the [h^+^] (given by the change in optical density at 625 nm) which is modified by changing the applied potential to the electrode, Fig. 5a. Full details of this experiment can be found alongside Fig. S15.† This experiment is analogous to the photoinduced absorption experiment (PIA) previously reported by Durrant and colleagues to identify the rate law for water oxidation on hematite by photogenerated holes.^15^ There they correlated the measured photocurrent for a hematite photoelectrode under strong anodic bias to the optically determined [h^+^] which was modulated by changing the light intensity. In Fig. 5b we also show the results of a PIA experiment carried out on the same hematite electrode.

In-line with the past PIA studies we find that both the dark and the photoelectrochemical water oxidation currents/rates depend strongly on [h^+^].^15^ For both the VIA and PIA experiments there is an approximately first-order dependence of *J*^wo^ on hole density (*α* = 1) at low h^+^ concentrations, which switches to third order (*α* = 3) at higher h^+^ concentrations, Fig. 5a and b. The transition between 1^st^ order and 3^rd^ order behaviour occurs at approximately the same hole density in the light and dark (∼2 h^+^ nm^−2^, based of electrode geometric surface area) and a *J*^wo^ of ∼0.03–0.04 e^−^ s^−1^ nm^−2^ (0.4 and 0.6 μA cm^−2^). This compares well to past PIA studies on planar hematite where a change in reaction order occurred at between ∼0.6 h^+^ nm^−2^ and a *J*^wo^ ∼ 0.07 e^−^ s^−1^ nm^−2^ (∼1 μA cm^−2^).^40^ On high aspect ratio photoelectrodes which achieve benchmark photocurrents the change in reaction order to *α* = 3 occurs at similar surface hole densities (∼1 h^+^ nm^−2^) to those seen here and the third-order dependence of *J*^wo^ on hole density has been shown to occur at ∼1 sun indicating that it is occurring on many leading photoelectrodes.^15^ Interestingly here at the highest water oxidation currents studied (*J*^wo^ > 0.3 (VIA), *J*^wo^ > 0.6 e^−^ s^−1^ nm^−2^ (PIA)) in both the dark and light we find that *α* ≠ 1 or 3 which has not been previously reported. Instead we find that the *J*^wo^*vs.* h^+^ density data in this region can be reasonably well fitted to a linear function with *α* ∼ 7–9 (Fig. 5a and b) and this will be discussed further below.

Fig. 5c allows us to make comparison between the VIA data and the EFISHG response as the [h^+^] changes (Fig. 3). There is a slight offset in the potentials of the reference electrode between the VIA and EFISHG experiment (*ca.* 20 mV) which is reasonable given the use of a pseudo reference (Ag wire) and the change in spectroelectrochemical cell. From Fig. 5c it can be seen that h^+^ accumulation increases at potentials where *J*^wo^ has a first or third-order dependence on h^+^ density (*α* = 1 or 3 from 0.575 to 0.675 V_Ag/Ag^+^_). The EFISHG experiment (Fig. 3) shows that this h^+^ accumulation leads to potential drop across the Helmholtz layer due to Fermi level pinning. If the rate limiting step of the oxygen evolution reaction involves ion or electron transfer at the electrode surface a change in Δ*ϕ*_H_ is expected to change *k*_wo_.^41,42^ Fig. S18† shows that when the water oxidation mechanism has a first or third-order dependence of *J*^wo^ on hole density, *k*_wo_ is approximately constant as the [h^+^] changes when *α* = 1 or 3. Therefore, we conclude that *k*_wo_ is independent of Δ*ϕ*_H_ during electrocatalytic water oxidation on hematite in the dark. This conclusion is in-line with the assignment of h^+^s to an Fe^IV^=O state, formed following Fe^III^–O(H) oxidation^43,44^ and DFT calculations on the photoelectrochemical mechanism of water oxidation on hematite that show for low Fe^IV^=O coverages (when *α* = 1) the rate determining step is the interaction of H_2_O with Fe^IV^=O, leading to O–O bond formation.^40^ At higher coverages (*α* = 3) the rate determining step is again O–O bond formation, this time due to nucleophilic attack of a water or hydroxide molecule at site where three h^+^ have accumulated.^16,40^ In both cases the rate determining step (O–O bond formation) does not involve a formal transfer of a charged species (H^+^, e^−^) to or from the solution and the reaction free energy would not be expected to be strongly dependent upon the potential drop across the Helmholtz layer as is seen in our studies.

The good agreement between the PIA and VIA data shows that water oxidation occurs by the same surface mechanisms in the dark and the light on these hematite electrodes. Ideally EFISHG experiments would also be carried out with the electrode under band gap illumination to directly assess the change in Δ*ϕ*_H_ during photohole accumulation, however these are extremely difficult to carry out and to interpret due to the presence (and trapping) of both photogenerated h^+^ and e^−^. The demonstration of a common mechanism during electrocatalytic (dark) and photoelectrochemical water oxidation indicates that the rate determining step is also likely to be the same and it is therefore reasonable to conclude that *k*_wo_ would also be expected to be independent of Δ*ϕ*_H_ under conditions where *J*^wo^ has a first or third-order dependence on hole density.

Finally, we discuss the VIA/PIA results measured at high current densities where a third order dependence of *J*^wo^ on h^+^ density no longer holds, Fig. 5. Instead, the data is better fitted to *α* ∼ 7 to 9, although there are considerable errors on these linear fits. This behaviour has not previously been reported on hematite. A possible cause could be direct water oxidation due to hole transfer from FTO *via* pinholes in the α-Fe_2_O_3_ layer. But we rule this out as the onset of water oxidation on FTO is significantly positive of that on α-Fe_2_O_3_ and Fig. S11† shows that at the potentials used in the VIA experiment the FTO is largely inactive. Instead, we propose that the water oxidation is still primarily occurring on hematite and that a further change in reaction mechanism for water oxidation on α-Fe_2_O_3_ has occurred at the highest *J*^wo^ measured. It is notable that the potential region where *α* ≠ 1 or 3 corresponds to that where the [h^+^] plateaus (>0.7 V_Ag/Ag^+^_, Fig. 5c), where the EFISHG experiment shows that the rate of change of *I*_SHG_ with applied potential becomes very small. One interpretation of this result is that the potential drop is now nearly completely over the Helmholtz layer as increases in *ϕ*Δ_SC_ are shown above to lead to large increases in *I*_SHG_. In this case a switch to a mechanism where the rate determining step is dependent on Δ*ϕ*_H_ may rationalise the behaviour in Fig. 5. However, a plot of *k*_wo_ for *α* = 8 at range of h^+^ densities does not show a clear correlation between h^+^ density/applied electrode potential (Fig. S18†) which suggests that the population model still holds. In this case the rate determining step of the oxygen evolution reaction under these conditions also does not involve ion or electron transfer. But as noted above we caution there is considerable error in the fit of the VIA and PIA data at the higher values of *J*^wo^ and further experiments are need to explore this interesting result.

## Conclusion

In conclusion our study demonstrates the applicability of EFISHG as a probe of the electric fields present at the SCLJ during electrochemical water splitting. Using complimentary UV/vis spectroelectrochemical measurements we have directly correlated changes in [h^+^] with changes in the interfacial electric fields determined from EFISHG. At potentials where surface h^+^ accumulation occurs, immediately prior to the onset of water oxidation, there is a shift in the potential distribution leading to a change in Δ*ϕ*_H_. Whilst simple absorbance measurements are able to identify the accumulation of holes in the electrode it is through the EFISHG that we are able to identify the impact of this on the potential distribution and fields across the interface. Despite the change in Δ*ϕ*_H_ we measured no significant change in the rate constant for water oxidation (*k*_wo_) at potentials where *J*^wo^ has a first or third-order dependence on h^+^ density. This indicates that the rate determining step in water oxidation on hematite at these lower current densities does not involve a formal transfer of a charge species and instead our results are consistent with O–O formation being the rate determining step. Here we have focused on the behaviour of semiconductor electrodes in the dark but with careful modelling, experiments of photoelectrodes under band gap illumination should be addressable. This combined with the ability to carry out EFISHG with a high spatial resolution (μm) offers an exciting future opportunity to provide a comprehensive understanding of the spatial and potential dependence of the SCLJ during photoelectrochemical water oxidation.

## Data availability

The ESI† contains full details of experimental procedures and supporting experimental data. The raw data associated with the figures in the main text can be found at no charge at https://doi.org/10.17638/datacat.liverpool.ac.uk/2158.

## Author contributions

Khezar H. Saeed: conceptualisation, data curation, formal analysis, investigation, writing – original draft, writing – review & editing. Dora-Alicia Garcia Osario: data curation, formal analysis, investigation, writing – review & editing. Chao Li: data curation, formal analysis, investigation, writing – review & editing. Liam Banerji: data curation, formal analysis, investigation, writing – review & editing. Adrian M. Gardner: conceptualisation, investigation, supervision, writing – review & editing. Alexander J. Cowan: conceptualisation, formal analysis, funding acquisition, investigation, supervision, writing – original draft, writing – review & editing.

## Conflicts of interest

There are no conflicts of interest to declare.

## Supplementary Material

SC-014-D2SC05628C-s001

## References

[cit1] Sivula K., Formal F. L., Grätzel M. (2011). ChemSusChem.

[cit2] Peter L. M. , in Photocatalysis: Fundamentals and Perspectives, RSC, 2016, pp. 1–28

[cit3] Hankin A., Bedoya-Lora F. E., Alexander J. C., Regoutz A., Kelsall G. H. (2019). J. Mater. Chem. A.

[cit4] Formal F. L., Sivula K., Gra M., Formal F. L., Sivula K., Grätzel M. (2012). J. Phys. Chem. C.

[cit5] Forster M., Cheung D. W. F., Gardner A. M., Cowan A. J. (2020). J. Chem. Phys..

[cit6] Pendlebury S. R., Wang X., Formal F. L., Cornuz M., Kafizas A., Tilley S. D., Grätzel M., Durrant J. R. (2014). J. Am. Chem. Soc..

[cit7] Pendlebury S. R., Cowan A. J., Barroso M., Sivula K., Ye J., Grätzel M., Klug D. R., Tang J., Durrant J. R. (2012). Energy Environ. Sci..

[cit8] Cowan A. J., Barnett C. J., Pendlebury S. R., Barroso M., Sivula K., Grätzel M., Durrant J. R., Klug D. R. (2011). J. Am. Chem. Soc..

[cit9] Pesci F. M., Cowan A. J., Alexander B. D., Durrant J. R., Klug D. R. (2011). J. Phys. Chem. Lett..

[cit10] Ma Y., Pendlebury S. R., Reynal A., Le Formal F., Durrant J. R. (2014). Chem. Sci..

[cit11] Corby S., Rao R. R., Steier L., Durrant J. R. (2021). Nat. Rev. Mater..

[cit12] Lewis N. S. (1998). J. Phys. Chem. B.

[cit13] Walter M. G., Warren E. L., Mckone J. R., Boettcher S. W., Mi Q., Santori E. A., Lewis N. S. (2010). Chem. Rev..

[cit14] Francàs L. , Mesa C. A., Pastor E., Le Formal F. and Durrant J. R., in Energy and Environment Series, ed. S. D. Tilley, S. Lany and R. van de Krol, Royal Society of Chemistry, Cambridge, 2018, pp. 128–162

[cit15] Formal F. L., Pastor E., Tilley S. D., Mesa C. A., Pendlebury S. R., Grätzel M., Durrant J. R. (2015). J. Am. Chem. Soc..

[cit16] Righi G., Plescher J., Schmidt F.-P., Campen R. K., Fabris S., Knop-Gericke A., Schlögl R., Jones T. E., Teschner D., Piccinin S. (2022). Nat. Catal..

[cit17] Mesa C. A., Steier L., Moss B., Francàs L., Thorne J. E., Grätzel M., Durrant J. R. (2020). J. Phys. Chem. Lett..

[cit18] Zhang S., Leng W. (2020). Nat. Chem..

[cit19] Peter L. , in Energy and Environment Series, ed. H.-J. Lewerenz and L. Peter, Royal Society of Chemistry, Cambridge, 2013, pp. 19–51

[cit20] Cummings C. Y., Marken F., Peter L. M., Tahir A. A., Wijayantha K. G. U. (2012). Chem. Commun..

[cit21] Peter L. M., Wijayantha K. G. U., Tahir A. A. (2012). Faraday Discuss..

[cit22] Klahr B., Gimenez S., Fabregat-Santiago F., Hamann T., Bisquert J. (2012). J. Am. Chem. Soc..

[cit23] Klahr B., Gimenez S., Fabregat-Santiago F., Bisquert J., Hamann T. W. (2012). Energy Environ. Sci..

[cit24] Shen Y. R. , The Principles of Nonlinear Optics, Wiley, New York, 1984

[cit25] Eisert F. , Elg A.-P. C. and Rosen A., in Laser Techniques for Surface Science III, SPIE, 1998, vol. 3272, pp. 7–14

[cit26] Hamkalo M., Fita P., Fedorynski M., Makosza M. (2018). Chem.–Eur. J..

[cit27] Ong S., Zhao X., Eisenthal K. B. (1992). Chem. Phys. Lett..

[cit28] Lee C. H., Chang R. K., Bloembergen N. (1967). Phys. Rev. Lett..

[cit29] Richmond G. L., Rojhantalab H. M., Robinson J. M., Shannon V. L. (1987). J. Opt. Soc. Am. B.

[cit30] Bian H., Guo Y., Wang H. (2018). Phys. Chem. Chem. Phys..

[cit31] Kautek W., Sorg N., Krüger J. (1994). Electrochim. Acta.

[cit32] Lantz J. M., Corn R. M. (1994). J. Phys. Chem..

[cit33] Aktsipetrov O. A., Baranova I. M., Grigor'eva L. V., Evtyukhov K. N., Mishina E. D., Murzina T. V., Chernyĭ I. V. (1991). Sov. J. Quantum Electron..

[cit34] Lantz J. M., Baba R., Corn R. M. (1993). J. Phys. Chem..

[cit35] Thämer M., Campen R. K., Wolf M. (2018). Phys. Chem. Chem. Phys..

[cit36] Jordan D. S., Hull C. J., Troiano J. M., Riha S. C., Martinson A. B. F., Rosso K. M., Geiger F. M. (2013). J. Phys. Chem. C.

[cit37] Troiano J. M., Jordan D. S., Hull C. J., Geiger F. M. (2013). J. Phys. Chem. C.

[cit38] Marusak L. A., Messier R., White W. B. (1980). J. Phys. Chem. Solids.

[cit39] Bard A. J., Bocarsly A. B., Fan F. R. F., Walton E. G., Wrighton M. S. (1980). J. Am. Chem. Soc..

[cit40] Mesa C. A., Francàs L., Yang K. R., Garrido-Barros P., Pastor E., Ma Y., Kafizas A., Rosser T. E., Mayer M. T., Reisner E., Grätzel M., Batista V. S., Durrant J. R. (2020). Nat. Chem..

[cit41] Nong H. N., Falling L. J., Bergmann A., Klingenhof M., Tran H. P., Spöri C., Mom R., Timoshenko J., Zichittella G., Knop-Gericke A., Piccinin S., Pérez-Ramírez J., Cuenya B. R., Schlögl R., Strasser P., Teschner D., Jones T. E. (2020). Nature.

[cit42] Boettcher S. W., Surendranath Y. (2021). Nat. Catal..

[cit43] Zandi O., Hamann T. W. (2016). Nat. Chem..

[cit44] Zandi O., Hamann T. W. (2015). Phys. Chem. Chem. Phys..

