# Supplementary material for: Monitoring interfacial electric fields at a hematite electrode during water oxidation
Source: Chem Sci. 2023 Feb 23;14(12):3182–9. doi: 10.1039/d2sc05628c (PMC10034152; doi:10.1039/d2sc05628c)
Supplement: SC-014-D2SC05628C-s001 [file SC-014-D2SC05628C-s001.pdf]

Electronic Supplementary Material (ESI) for Chemical Science.
This journal is © The Royal Society of Chemistry 2023

Supporting Information

Monitoring of Interfacial Electric Fields at a Hematite Electrode During Water Oxidation

Khezar H. Saeed[1,2], Dora-Alicia Garcia Osorio[1], Chao Li[1], Liam Banerji,[1] Adrian M. Gardner[1,3] and Alexander J. Cowan[1*]

1. Department of Chemistry and Stephenson Institute for Renewable Energy, University of Liverpool, Liverpool UK, acowan@liverpool.ac.uk

2. Current address: Department of Chemistry, Aarhus University, Aarhus, Denmark

3. Early Career Laser Laboratory, University of Liverpool, Liverpool UK

1. Experimental details:

Electrodeposition of hematite:

The electrodeposition procedure is adapted from Zandi et al.[1] FTO electrode substrates (NSG-Pilkington, TEC-15) are cleaned by successive sonication in acetone, then ethanol, then water (10 mins each). $FeCl_2$ (0.1 M, 20 ml) is adjusted to pH ~4 with HCl and held at 60 °C with gentle stirring. The cleaned FTO substrate (~1x1 cm submerged in electrolyte) is held at 1.2 V vs Ag wire (with a Pt wire counter electrode) until 0.5 C charge passed. The resulting films are gently rinsed with water, then annealed at 700 °C (ramp rate not controlled) in air for 1 hour and allowed to cool to room temperature in the furnace. Under these annealing conditions mobile ions from the underlying substrates (usually reported as Sn from the FTO, but possibly also Na from the glass) are incorporated into the hematite as dopants. See Table S1 further below for an approximation of the dopant density of the films used here.

In situ electrochemical SHG spectroscopy:

An overview of the SHG experimental layout can be seen in Figure S1. Around 1 W of the output of a PHAROS-PH1-SP (Light Conversion, 1030 nm, 10 kHz, 10 W, 170 fs pulse duration) laser system is used to pump a visible OPA (ORPHEUS) to generate 800 nm light. Gold mirrors are used to direct the light to the sample position, through a halfwave plate and polariser (Thorlabs) and focused onto the sample position to a beam diameter of around 500 µm. The polariser and halfwave plate are oriented to give ~2 µJ/pulse p-polarised 800 nm light at the sample. A 450 nm long pass filter (Thorlabs FEL0450) was placed before the sample to remove the residual SHG response from the quartz in the waveplate (quartz has no centre of symmetry in its crystal structure so can be bulk active for 2nd order nonlinear processes like SHG). UV-enhanced aluminium mirrors are used to direct the beam after the sample as they have better reflectivity at the shorter SHG wavelength of 400 nm. A series of 550 nm short pass filters (Thorlabs FESH0550) are included to filter out residual 800 nm light, before the SHG light is focused onto the slits of a spectrograph (Andor, Kymera) and detected on a CCD camera (Andor, iDus).

Electrode samples are mounted in a custom electrochemical cell (Figure S2) that is 3D-printed in acrylonitrile butadiene styrene. An analogue potentiostat (Whistonbrook Technologies Ltd.) is used to synchronise SHG data acquisition and electrochemical measurements using a TTL trigger pulse and a custom LabView program. More details on data processing, including discussion of smoothing algorithms for the first derivative calculations can be found accompanying Figure S13.

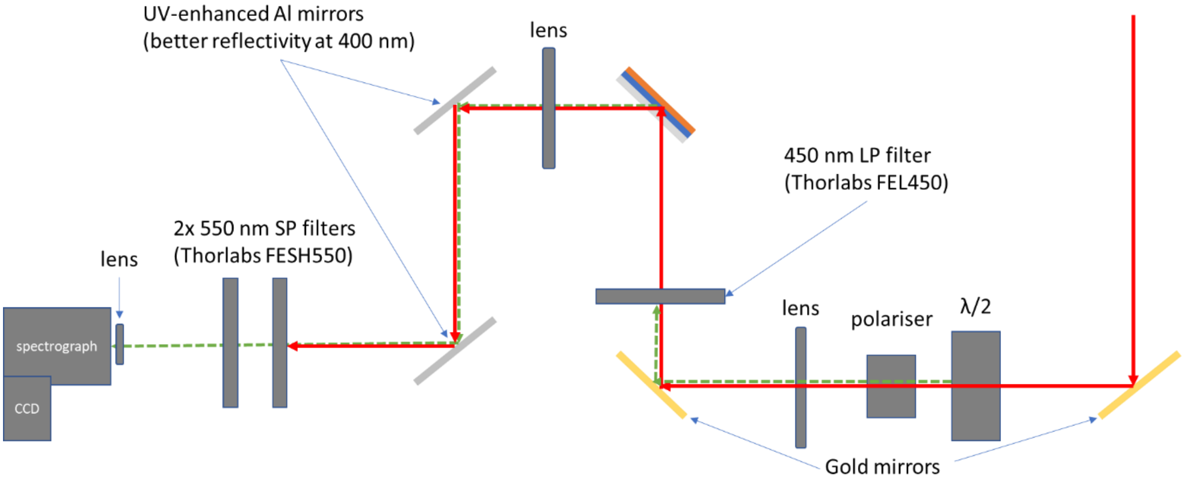

Figure S1: Diagram of beam layout for SHG experiments, where the red arrows represent the 800 nm beam-path, green arrows represent the SHG beam-path.

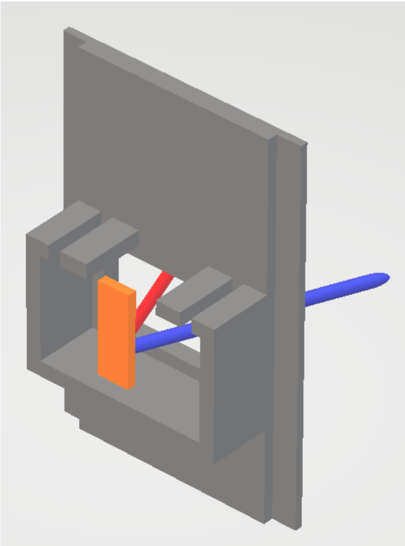

Figure S2: Spectroelectrochemical cell design and experimental geometry. The cell design contains a wide aperture for rectangular glass window that allows 800 nm light to reach the sample and reflected 400 nm SHG light to leave the cell. Three openings are included in the top of the cell to allow access

for working and counter electrodes as well as a contact for the working electrode. The hematite working electrode is mounted to a plastic "lid" (not shown here for clarity) which holds the electrode in place and is fixed to the cell body with epoxy.

A potential concern in the SHG experiment is that the steady-state carrier population is significantly changed by either the 800 nm incident light (through multiphoton absorption or via the population of sub-band gap states) or as a result of band gap excitation by the 400 nm photons from SHG. As noted in the main text the current-voltage response of the α-Fe_2O_3 electrode was measured whilst being excited by the 800 nm laser using identical conditions to those in the SHG experiment and no photocurrent was detected (see Figure S8 below). This is in line with expectations based on the absorption coefficients of the α-Fe_2O_3 electrode and the known photoactivity (incident photon to current efficiency, IPCE) behaviour of this type of electrode. At 800 nm the absorption coefficient is *ca.* 1 x 10^3 cm^{-1},[2] so photon absorption can occur via the presence of defect states however we are unaware of any reports where 800 nm excitation has led to the generation of long-lived charges and a detectable photocurrent (for example see references [1,3,4] which are typical for the field and show zero photocurrent at excitation wavelengths greater than 600 nm). 400 nm photons can lead to band gap excitation of α-Fe_2O_3 but it is possible to estimate the steady-state carrier concentration as a result of SHG and we find this to be several orders of magnitude lower than N_d (~10^{19} cm^{-3}, see table S1 below). We arrive at this conclusion as:

800 nm incident power = 25 mW = 1 x 10^{17} photons s^{-1}

The efficiency of SHG will be dependent on multiple factors including amongst others the electrolyte and the concentration used, the nature of the FTO/Fe_2O_3 interface and the applied potential. We are unaware of reported values for SHG efficiencies for a α-Fe_2O_3 aqueous interface but a review of the literature indicates that quantum efficiencies on the order of 10^{-6} are considered a reasonable upper estimate here.[5]

This gives rise to a maximum rate of 400 nm generation of 1 x 10^{11} photons s^{-1}

From the beam diameter (500 μm) and sample thickness (*ca.* 150 nm, see Figure S5) and assuming complete absorption of the 400 nm SHG (which we recognise is an over-estimate/worst case scenario) we arrive at an estimated maximum carrier generation rate of 3.4 x 10^{18} s^{-1} cm^{-3}. Fast transient absorption spectroscopy has shown that following band gap excitation with fs pulses there is significant carrier-recombination on the ps-ns timescale even at low excitation intensities even when the α-Fe_2O_3 sample is positively biased.[6] The majority of the carriers are found to have decayed within 100 μs. This observed fast recombination is in-line with the maximum IPCE reported for the α-Fe_2O_3 sample used here which is *ca.* 10-12% at 400 nm[1] and we would anticipate that the steady-state change in carrier density is on the order of 10^{17} cm^{-3}, approximately two orders of magnitude below N_d in the dark.

UV-Vis absorption spectroscopy:

All UV-Vis spectra were recorded on a Shimadzu UV-2600 spectrophotometer, used in transmission mode with the internal detector. Note that the high annealing temperature had caused the underlying glass structure of the hematite electrode to lose some transparency across the spectrum due to scattering. The data in Figure 2 (main text) is shown as a % transmission relative to air as no suitable background sample (*e.g.* bare FTO or annealed bare FTO) could be used due to this scattering. The in-situ surface hole concentration measurements (Figure 3, main text) were also carried out on the same spectrometer, this time in kinetics mode (at 625 nm) during a linear sweep measurement. The hematite electrode is mounted in a quartz cuvette with a Pt wire counter electrode and Ag wire pseudo-reference electrode. Steady-state surface hole concentration measurements (Figure 5, main text) were carried out on a custom-built photo-induced absorption spectrometer, see Figure S15 for more details.

2. Characterisation data of the α-Fe2O3 electrodes

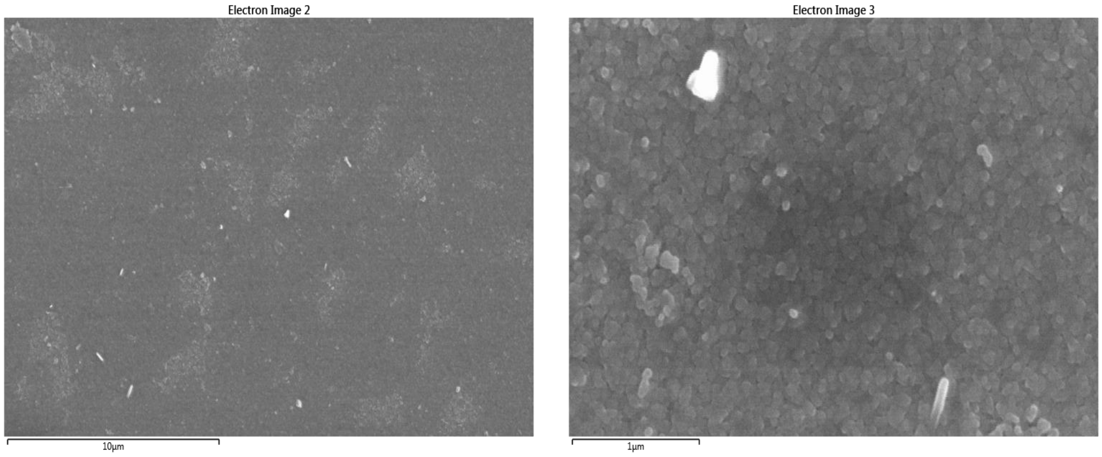

Figure S3: SEM images of the electrodeposited hematite films used throughout this study. Scale bars correspond to 10 µm (left) and 1 µm (right).

We note that some areas close to the edge of the electrodeposited films exhibit fissures, as shown in the image in Figure S4. SEM EDX mapping shows Fe inside the fissures, suggesting that the hematite layer is present across the entirety of the exposed surface.

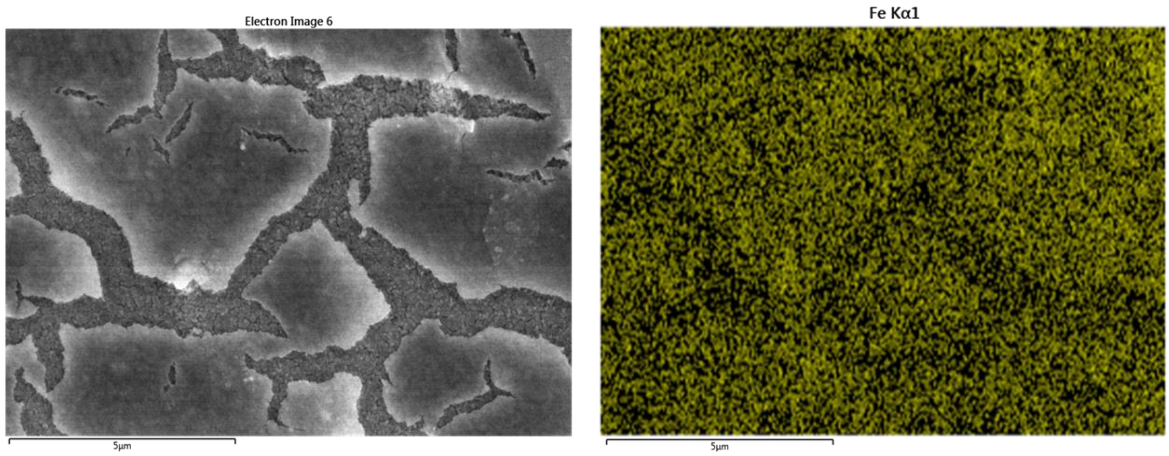

Figure S4: SEM image of the fissures found at the edges of the electrodeposited hematite films used throughout this study (left) and SEM EDX analysis showing the presence of iron in the gaps between the fissures (right). Scale bars correspond to 5 μm.

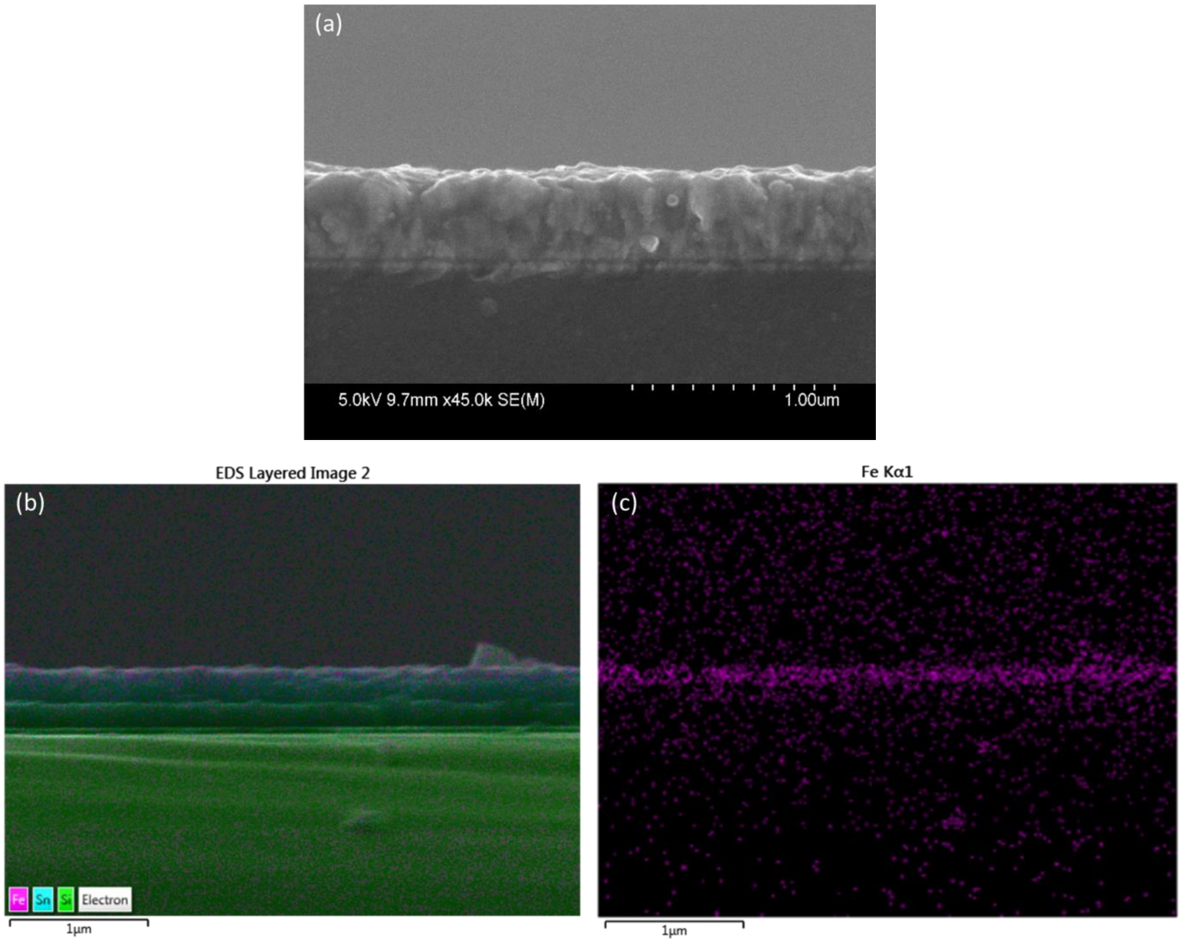

Figure S5: (a) Cross sectional SEM image of the film and SEM EDX analysis of the hematite film cross section showing (b) Fe (pink), Sn (blue) and Si (green) and (c) Fe only, giving an approximate hematite layer thickness of 150 nm.

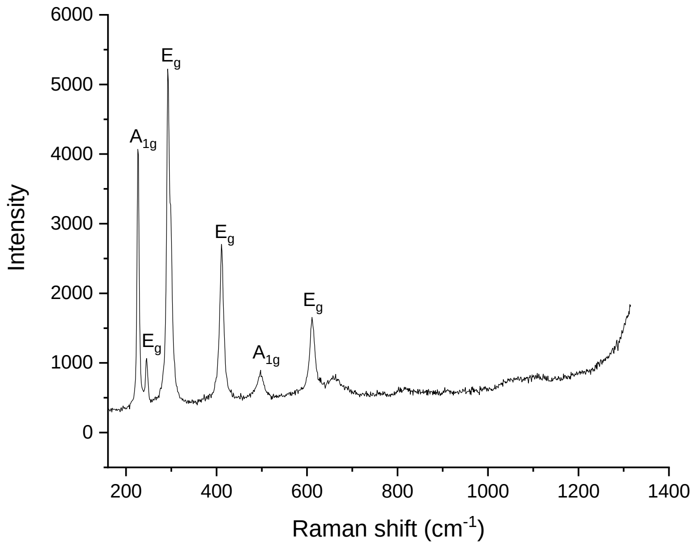

Figure S6: Raman spectrum (recorded on Renishaw InVia microscope with 633 nm laser) of the electrodeposited hematite films used throughout this study. Peak assignments to the A_{1g} and E_g modes of crystalline hematite are made based on comparison to Marshall et al.[7]

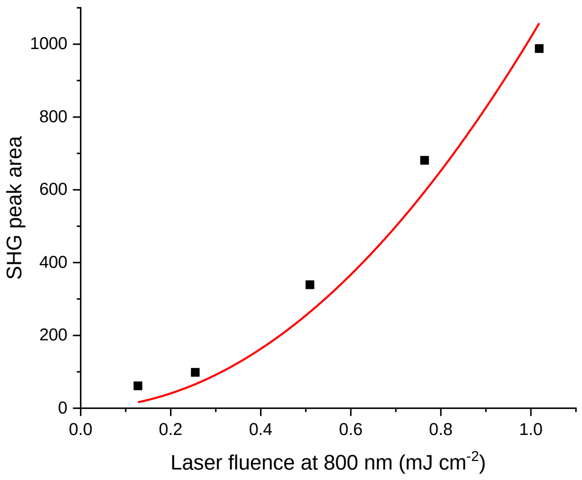

Figure S7: Laser fluence (at 800 nm) vs. SHG peak area for the hematite electrode used throughout with a quadratic fit in red, confirming the observed signal is a second order process (as expected for second harmonic generation).

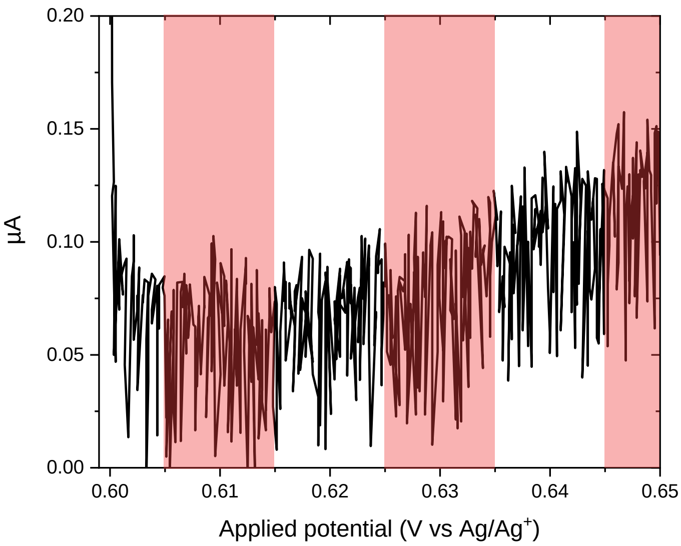

Figure S8: Slow linear sweep voltammogram of α-Fe_2O_3 electrode in 0.1 M NaOH at 0.05 mV s^{-1} under chopped laser illumination (800 nm, 25 mW), where red shaded periods indicate illumination. The lack of any substantial photocurrent under illumination indicates minimal absorption of both 800 nm incident light and generated second harmonic light at 400 nm.

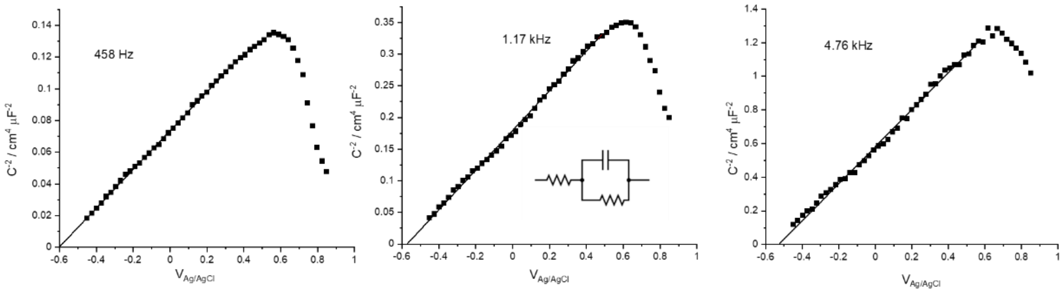

Figure S9: MS analysis of the α-Fe_2O_3 electrode in 0.1 M NaOH. MS analysis was carried out using the equivalent circuit diagram shown in the inset.

We find that the intercept is frequency dependent (-0.4 to -0.6 V between 89 and 4.76 kHz). The limitations of a Mott Schottky (MS) analysis of α-Fe_2O_3 have been discussed in detail elsewhere.[8] In particular it is important to recognise that the measured capacitance has contributions from both the Helmholtz layer and space charge layer capacitance (C_{SC} and C_H respectively, Eq S1).

$$\frac{1}{C_{interface}} = \frac{1}{C_{SC}} + \frac{1}{C_H} \qquad \text{Eq. S1}$$

Typically, it is assumed that $C_{SC} << C_H$ but as we show in the main text the presence of surface states leads to an applied potential dependence of the distribution across the interface and this gives rise to the non-linearity (and frequency dependence) of the plots of $\frac{1}{C_{interface}^2}$ vs. applied potential seen in Figure S9. Therefore, from this data alone it is not possible to determine how $\phi\Delta_H$ vs. $\phi\Delta_{SC}$ is varying and the measured intercepts only give an estimate of the flat band potential. Following recent guidelines[8] we have therefore carried out a 2nd measurement of the flat band potential by assessment of the chopped photocurrent (Figure S10). This shows a transition between anodic and cathodic photocurrent spikes at *ca.* -0.46 V. Referencing the Ag/Ag$^+$ pseudo reference electrode to a Ag/AgCl we measure a potential difference of +0.1 V but as we note in the main text the exact measured potential versus Ag/Ag$^+$ is found to be sensitive to the cell geometry used.

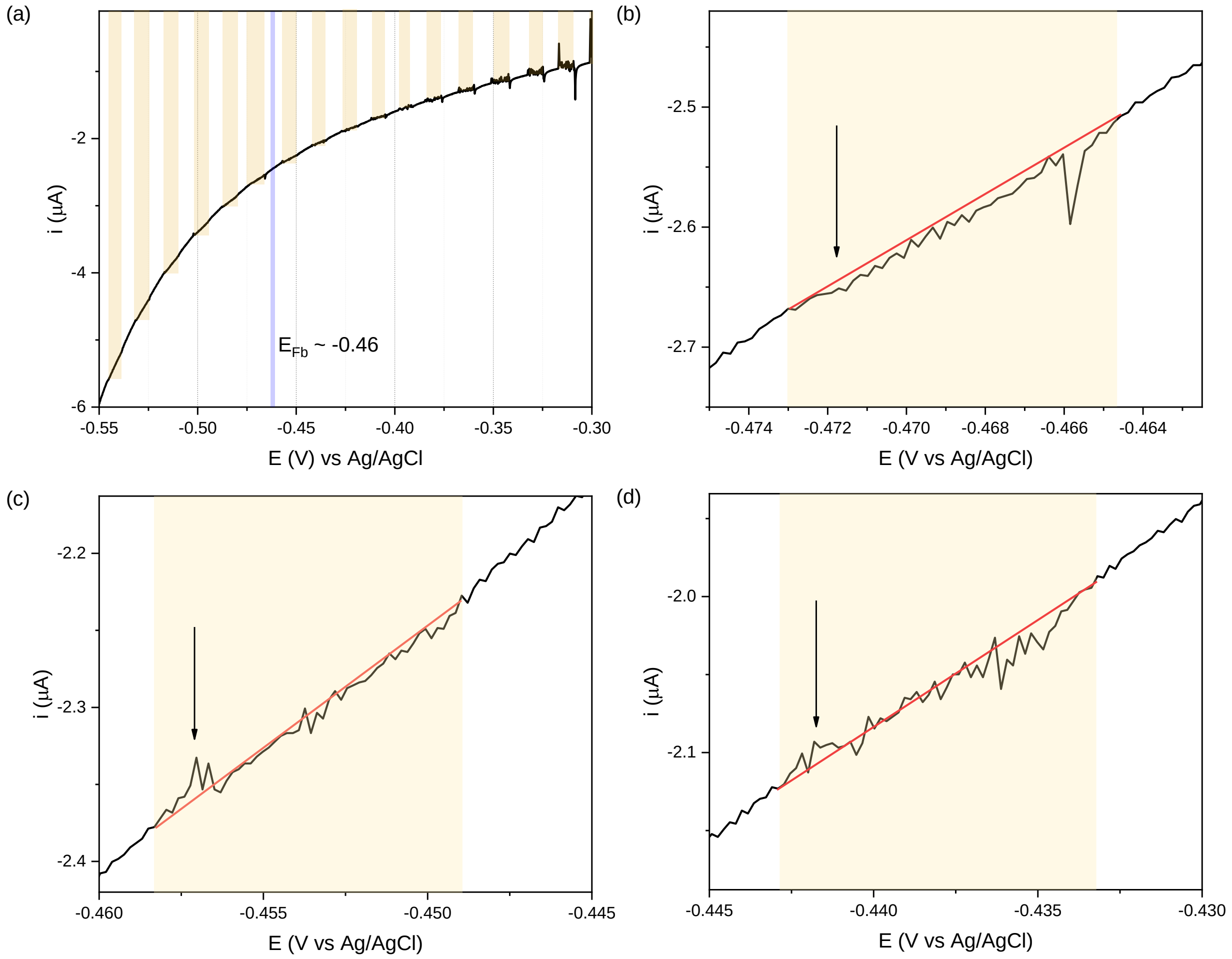

Figure S10: (a) Chopped photocurrent of the α-Fe_2O_3 electrode in 0.1 M NaOH. Yellow bars represent periods of illumination and the blue line indicates the potential where there is a transition between

anodic and cathodic photocurrent spikes. (b-d) Zoomed-in regions of the chopped photocurrents in (a), with arrows indicating the transient photocurrent spike and a straight red line drawn to guide the eye for the sloping baseline. This spike in photocurrent changes sign between panels (b) and (c), indicating the flat band potential lies between these two potential windows (approx. -0.46 V vs Ag/AgCl). Panel (d) is included to show that this spike remains positive at more positive potentials.

Using the chopped photocurrent measurements to approximate the flat band potential (V_{fb}), the slopes of the Mott-Schottky plots at the three different frequencies are used to approximate the dopant density (N_D) using the Mott-Schottky equation:

$$\frac{1}{C^2} = \frac{2}{\varepsilon\varepsilon_0 A^2 e N_D}\left(V - V_{fb} - \frac{k_B T}{e}\right) \qquad \text{Eq. S2}$$

And by extension the space charge layer width (W_{SC}):

$$W_{SC} = \left(\frac{2\varepsilon\varepsilon_0}{eN_D}\left(V - V_{fb}\right)\right)^{\frac{1}{2}} \qquad \text{Eq. S3}$$

Where C is the differential capacitance, ε_0 is the permittivity of free space, A is the surface area of the electrode, e is the elementary charge, k_B is the Boltzmann constant, T is the temperature, ($V – V_{fb}$) is the difference between the relevant potential and flat band potential (to understand the behaviour under operating conditions a value of 1.23 V vs RHE is used here for V and V_{fb} is 0.505 V vs RHE under the pH 13 operating conditions of the cell), ε is the dielectric constant of hematite (typically used values for the dielectric constant of hematite range between 12 and 120, a value of 39 was used here for a film of average crystal packing density based on the calculations of Walsh *et al.*[9])

Table S1: Approximations of the dopant density and space charge layer width (under operating conditions at 1.23 V vs RHE) using the above Mott-Schottky slope the and the flat band potential determined by chopped photocurrent.

Freq (Hz)	Slope (C^{-2}/V)	Dopant density (cm^{-3})	W_{SC} (nm)
458	0.1211	2.99E+19	8.54
1167	0.3129	1.16E+19	13.73
4763	1.0971	3.30E+18	25.70

3. The potential dependence of EFISHG response

Equation 6 from the main manuscript is reproduced here (Equation S4)

$$P_{SHG} \approx \boldsymbol{E}(\omega)\boldsymbol{E}(\omega)\left[\left(\chi^{(2)}(\phi) + \chi_{SC}^{(3)}(\phi)\sqrt{\Delta\phi_{SC}}\right]\right. \quad \text{Eq. S4}$$

Equation S4 holds for when the potential drop is changing primarily across the space-charge layer with negligible change in the potential drop across the double layer ($\Delta\phi_{DL}$). If the potential is also dropping across the double layer then we can approximate that P_{SHG} will be

$$P_{SHG} \approx \boldsymbol{E}(\omega)\boldsymbol{E}(\omega)\left[\left(\chi^{(2)}(\phi) + \chi_{SC}^{(3)}(\phi)\sqrt{\Delta\phi_{SC}} + \chi_{DL}^{(3)}\Delta\phi_{DL}\right]\right. \quad \text{Eq. S5}$$

In Equation S5 we have approximated the field to be constant over the electrical double layer, giving a linear relationship in-line with several past studies.[10,11] This is reasonable as in this work we have carried out experiments only at high electrolyte concentrations (0.1 M NaOH) and potential drop within the electrolyte is primarily across the Helmholtz layer.

Expansion of equation S5, and considering that the change in I_{SHG} is proportional to the square of P_{SHG} gives rise to

$$I_{SHG} \propto \boldsymbol{E}(\omega)^4\left[\left|\chi^{(2)}\right|^2 + \left|\chi_{SC}^{(3)}\right|^2\Delta\phi_{SC} + \left|\chi_{DL}^{(3)}\right|^2\Delta\phi_{DL}{}^2 + 2Re\left[\chi^{(2)}\chi_{SC}^{(3)}e^{i\theta_1}\right]\sqrt{\Delta\phi_{SC}} + 2Re\left[\chi^{(2)}\chi_{DL}^{(3)}e^{i\theta_2}\right]\Delta\phi_{DL} + 2Re\left[\chi_{SC}^{(3)}\chi_{DL}^{(3)}e^{i\theta_3}\right]\sqrt{\Delta\phi_{SC}}\Delta\phi_{DL}\right] \quad \text{Eq. S6}$$

Where the exponential terms account for the phase differences between the interfacial $\chi^{(2)}$ contribution and the $\chi^{(3)}$ contributions from the space charge and double layer structure. Without heterodyne detection it is difficult to extract these relative phases and gain a quantitative understanding of their contributions. However, through a consideration of different scenarios (see below) we demonstrate that the observation of the linear change in I_{SHG} can be used to indicate that the potential drop is primarily across the space charge layer between -0.2 to -0.05 and 0.2 to 0.6 $V_{Ag/Ag+}$.

<u>Case 1</u><u>: $\Delta\phi_{SC}$</u> >> $\Delta\phi_{DL}$

$$I_{SHG} \propto \mathbf{E}(\omega)^4\left[\left|\chi^{(2)}\right|^2 + \left|\chi_{SC}^{(3)}\right|^2\Delta\phi_{SC} + \cancel{\left|\chi_{DL}^{(3)}\right|^2\Delta\phi_{DL}{}^2} + 2Re\left[\chi^{(2)}\chi_{SC}^{(3)}e^{i\theta_1}\right]\sqrt{\Delta\phi_{SC}} + \cancel{2Re\left[\chi^{(2)}\chi_{DL}^{(3)}e^{i\theta_2}\right]\Delta\phi_{DL}} + \cancel{2Re\left[\chi_{SC}^{(3)}\chi_{DL}^{(3)}e^{i\theta_3}\right]\sqrt{\Delta\phi_{SC}}\Delta\phi_{DL}}\right]$$

In this scenario we can strike out all terms from Eq 6 which depended on $\Delta\phi_{DL}$ as we assume this value to be negligible. This leaves the potential dependent terms highlighted in yellow. From this we can see the I_{SHG} response will be linear with applied potential if $\chi^{(2)} < \chi_{SC}^{(3)}$ as the $\left|\chi_{SC}^{(3)}\right|^2\Delta\phi_{SC}$ term dominates.

<u>Case</u> <u>2: $\Delta\phi_{SC}$</u> << $\Delta\phi_{DL}$

$$I_{SHG} \propto \mathbf{E}(\omega)^4 \Big[\left|\chi^{(2)}\right|^2 + \cancel{\left|\chi^{(3)}_{SC}\right|^2 \Delta\phi_{SC}} + \left|\chi^{(3)}_{DL}\right|^2 \Delta\phi_{DL}{}^2 + \cancel{2\mathrm{Re}\left[\chi^{(2)}\chi^{(3)}_{SC} e^{i\theta_1}\right] \sqrt{\Delta\phi_{SC}}}$$

$$+ 2\mathrm{Re}\left[\chi^{(2)}\chi^{(3)}_{DL} e^{i\theta_2}\right] \Delta\phi_{DL} + \cancel{2\mathrm{Re}\left[\chi^{(3)}_{SC}\chi^{(3)}_{DL} e^{i\theta_3}\right] \sqrt{\Delta\phi_{SC}}\Delta\phi_{DL}} \Big]$$

In this scenario we can strike out all terms which depended on $\Delta\phi_{DL}$ as we assume this value to be negligible. This leaves the potential dependent terms highlighted in yellow.

If $\chi^{(2)} \ll \chi^{(3)}_{DL}$ then $\left|\chi^{(3)}_{DL}\right|^2 \Delta\phi_{DL}{}^2$ dominates and will observe a quadratic response in I_{SHG} with applied potential

If $\chi^{(2)} \gg \chi^{(3)}_{DL}$ then the term $2\mathrm{Re}\left[\chi^{(2)}\chi^{(3)}_{DL} e^{i\theta_2}\right] \Delta\phi_{DL}$ may contribute, showing a linear depdence with applied potential. However, under these conditions we would anticipate that the $\left|\chi^{(2)}\right|^2$term would dominate and for the I_{SHG} to be largely potential independent.

If $\chi^{(3)}_{SC} \gg \chi^{(3)}_{DL}$ then the term $\left|\chi^{(3)}_{DL}\right|^2 \Delta\phi_{DL}{}^2$ will be negligible and similarly we would anticipate any potential dependent contribution from the $2\mathrm{Re}\left[\chi^{(2)}\chi^{(3)}_{DL} e^{i\theta_2}\right] \Delta\phi_{DL}$ term to be very small. The term $2\mathrm{Re}\left[\chi^{(3)}_{SC}\chi^{(3)}_{DL} e^{i\theta_3}\right] \sqrt{\Delta\phi_{SC}}\Delta\phi_{DL}$ is also considered but as $\Delta\phi_{DL} \gg \Delta\phi_{SC}$ and as $\chi^{(3)}_{DL}$ is minimal we do not believe this would give rise to the observed linear changes in I_{SHG} with applied potential.

Therefore, we conclude that a large (relative to the potential indepdendent SHG response) linear change in I_{SHG} with applied potential at potentials positive of flat-band is indicative of $\Delta\phi_{SC} >> \Delta\phi_{DL}$. This is in agreement with past SHG studies on related materials (TiO_2) that reached the same conclusion.[12,13] The conclusion that the linear change in I_{SHG} between 0.2 and 0.6 V is due to $\Delta\phi_{SC} >> \Delta\phi_{DL}$ is further supported by the photocurrent response recorded in the main text (figure 2) which rises steeply in this potential region before plateauing at ~0.5 V (vs. Ag/AgCl, ca. 0.6 V Ag/Ag^+). It is widely accepted that the increase in photocurrent with applied potential is due to the increased charge separation efficiency which occurs as a result of the electric field across the space charge layer. This requires $\Delta\phi_{SC} \neq 0$ and the only other scenario we can identify which may give rise to a linear response in I_{SHG} requires $\Delta\phi_{SC} << \Delta\phi_{DL}$ which does not appear compatible with the photocurrent response.[14]

4. SHG response of FTO vs Fe_2O_3 interface:

As the potential is initially made more positive in both panels in Figure S11, I_{SHG} increases at a roughly constant rate, in line with their being a continuous increase in $\mathbf{E}_{DC}$. For an ideally behaving n-type semiconductor surface in an aqueous electrolyte it is well understood that as the potential of the electrode is made more positive the majority of the potential drop occurs across the space charge region and hence E_{SC} will increase and this is reflected in the strong linear potential dependence of the SHG

response between -0.2 and +0.4 V in Figure S11a. Conversely, both the initial magnitude (peak area of ~ 900 for FTO vs. 3000 for α-Fe_2O_3) and relative change (~10% increase for FTO vs. 230% for α-Fe_2O_3) of the FTO response over the same potential range are smaller (Figure S11b). The high conductivity of FTO under dark conditions would prevent the formation of a space charge layer, leading to a system dominated by the interfacial response ($\chi^{(2)} >> \chi^{(3)}$), which could explain this lower signal as there is minimal enhancement from the static electric field.

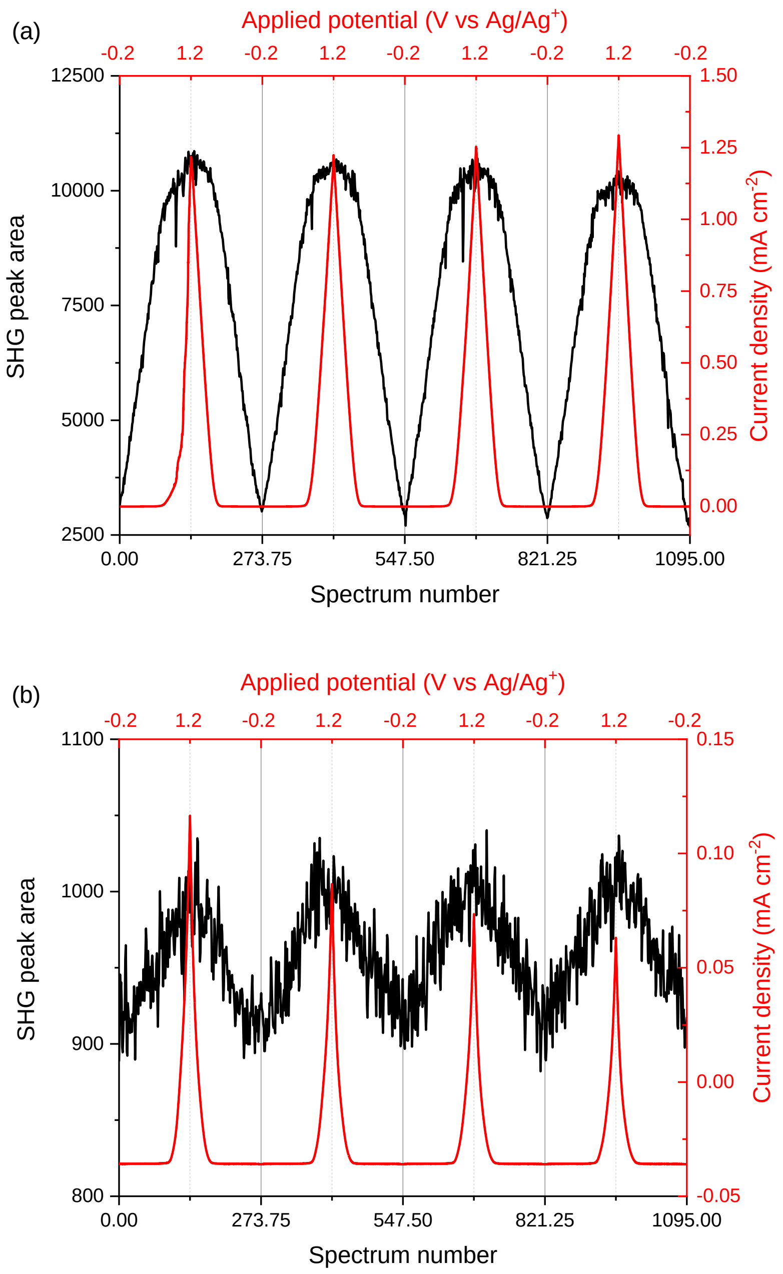

Figure S11: Integrated SHG peak area (λ_{SHG} = 400 nm, based on incident light at 800 nm at 25 mW) on α-Fe_2O_3 (a) and FTO electrodes (b) over 4 CV cycles at 2 mV s^{-1} in 0.1 M NaOH. Simultaneous current response shown in red.

5. Effect of ionic strength:

For data presented in the main text we used 0.1 M NaOH as the electrolyte in order to operate at the relevant (active) pH values used for hematite, and to avoid substantial local pH changes which could occur during water oxidation if a lower pH were to be used. Experiments at the same pH (0.1 M NaOH) but a higher ionic strength (by adding $NaClO_4$ as a supporting electrolyte to reach 2 M ionic strength) were carried out and are overlaid below (Figure S12a). In the raw data there does not seem to be an appreciable difference between the two different ionic strengths. The inconsistent dropouts in the 2 M data due to bubbles stuck to the surface limited further analysis (such as calculating d[SHG]/dV) without manually removing the dropouts which may introduce an unnecessary level of human bias. At 0.1 M NaOH the Debye length is approximately 1 nm (Figure S12b) and it is typically assumed that for the potential to drop across the space charge layer, the width of this layer would need to be similar to or less than the Debye length. In table S1 we estimate the width of the space charge layer to be > 8.5 nm , therefore further increasing the electrolyte concentration, which will further decrease the Debye length to < 1 nm, is not be predicted to change the distribution of the potential drop across the interface, in-line with our data in Figure S12b.

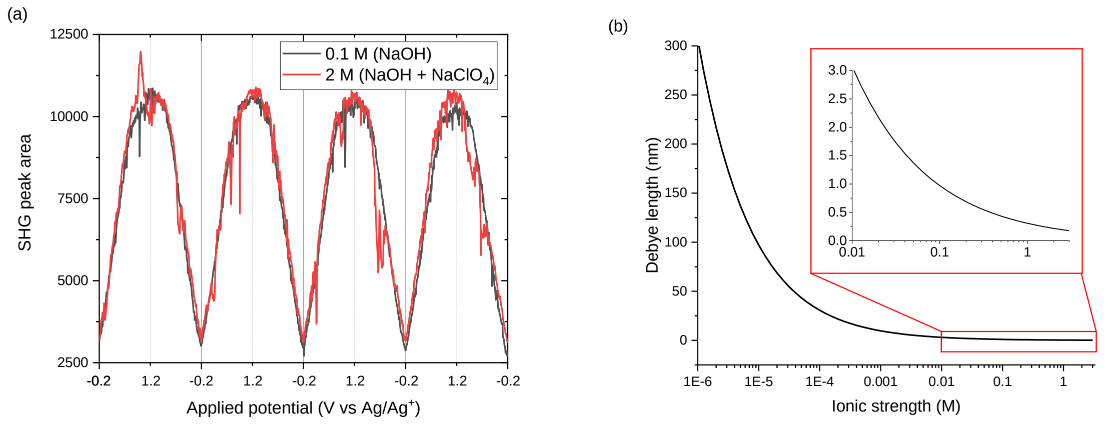

Figure S12: (a) Integrated SHG peak area (λ_{SHG} = 400 nm, based on incident light at 800 nm at 25 mW) on α-Fe_2O_3 over 4 CV cycles at 2 mV s^{-1} in 0.1 M NaOH or 0.1 M NaOH adjusted to an ionic strength of 2 M with $NaClO_4$. (b) Debye length as a function of ionic strength of solution, assuming purely monovalent cations. Calculated using $\lambda_D = \sqrt{\frac{\varepsilon_0 \varepsilon_r k_b T}{2 N_A c e^2}}$, where c is the ionic strength of the solution (in mol m^{-3}), T was set as 298 K and ε_r is the dielectric constant (a value of 80 was used here for water) and the rest of the terms are standard constants.

6. SHG data processing:

This section describes the treatment of the SHG data from the output of the spectrometer to the analysis of the first derivatives. Each voltage sweep experiment results in a set of spectra over time containing a single peak at 400 nm, corresponding to the SHG signal. The integrated peak areas can be plotted against the corresponding potential values during the voltage sweep, as shown in Figure 3, since the start of both electrochemical and spectroscopic experiments are synchronised with a TTL trigger pulse. As detailed in the main text and in the discussion above, the gradient of the potential-dependent SHG response can provide insight into the relative contributions of the second and third order susceptibilities to the observed SHG signal. Thus, analysis of these gradients by taking the first derivative of the SHG response with respect to applied potential is a useful procedure to aid our understanding of the system. Conventionally this kind of analysis requires pre-smoothing of experimental data to reduce noise in the resulting first derivative data. The default option for this smoothing in OriginLab (and many other code libraries) is the Savitzky-Golay algorithm, which has been shown to produce excessively noisy data and has issues with boundary artifacts.[15] An alternative smoothing algorithm was proposed by Schmid *et al.* (denoted as Modified Sinc) which improves on these deficiencies.[15] Both algorithms were applied to the data from Figure 3 in the main article and comparisons between the two smoothing methods is shown in Figure S13 below. The data smoothed using the Modified Sinc method appears to better match the experimental data (black lines in Figure S13a), especially around -0.2 V where the Savitzky-Golay smoothed data starts to deviate significantly from the experimental data. This deviation could lead to artefacts in the derivative data, as shown towards the edges of Figures S13b & S13c. Based on these comparisons all derivative data shown in the main article used the Modified Sinc algorithm provided by Schmid *et al.* (with degree = 2 and kernel halfwidth =60) prior to calculating the first derivative.[15]

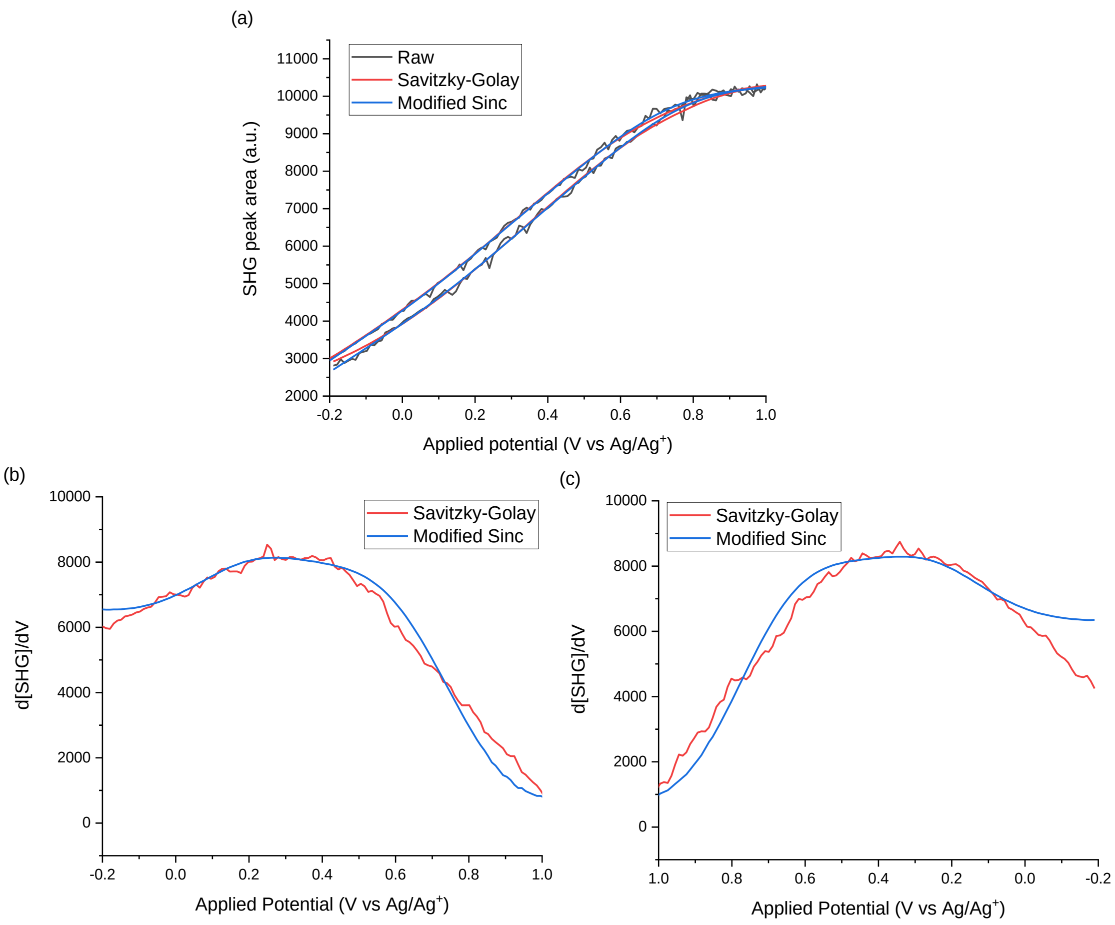

Figure S13: (a) Comparison of different smoothing algorithms on the experimental SHG peak area data from Figure 3 in the main text. Derivatives for the forward (b) and reverse (c) sweeps calculated from the smoothed data. Parameters for the Modified Sinc were degree = 2 and kernel halfwidth = 60 and for Savitzky-Golay, order = 2 and smoothing window = 60.

7. Steady-state surface hole concentration measurements

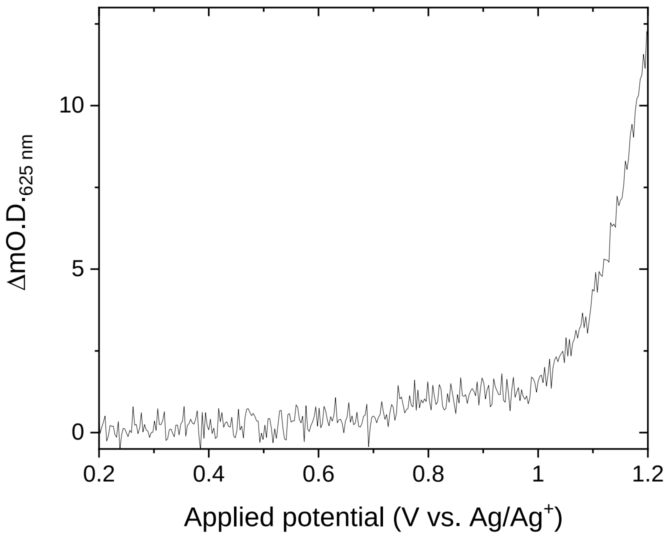

Figure S14: Change in optical density at 625 nm during a 2 mV s^{-1} LSV measurement over an extended voltage range. Following an initial rise in hole density (indicated by an increase in optical density) around 0.7 V we find the hole population plateaus between ~0.8 and 1.0 V. At > 1.0 V there is a sharp rise in the electrodes optical density at 625 nm which may indicate that it is becoming metallic. However we caution that at > 1.0 V the higher rate of oxygen evolution can lead to bubble formation complicating optical measurements.

The data shown in Figure 5 in the main text result from optical absorbance measurements under steady-state conditions either during illumination (PIA) or during a step change in applied bias (VIA). Both experiments were carried out on a custom-built spectrometer based on the output of a 75 W xenon lamp (PTI-A1010B). This light is passed through a monochromator (PTI) to generate the 625 nm probe beam, which is sent through a collimating lens and a focussing lens onto the sample area. Transmitted light through the sample is then re-collimated and focussed through a second monochromator, which includes additional 400 nm longpass filters (2x FEL400, Thorlabs) before hitting a reverse-biased avalanche photodiode. The raw voltage output of this photodiode is passed through a low-noise pre-amplifier (Stanford Research Systems SR560) and received by the LabView software through a DAQ card (National Instruments, NI-USB-6211) interface.

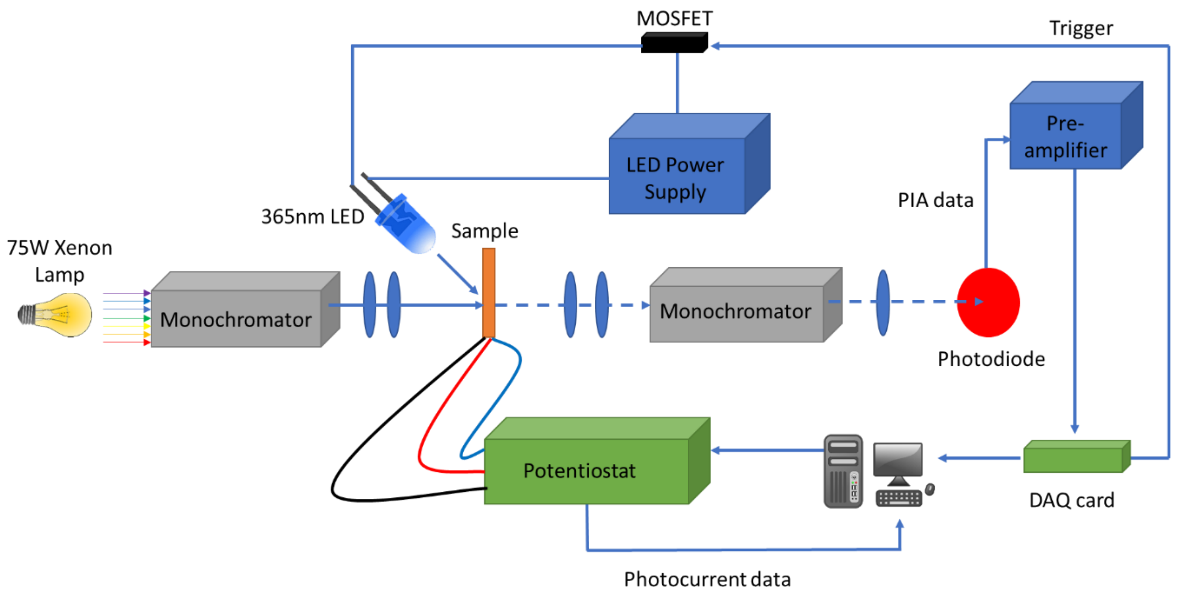

Figure S15: Schematic diagram of the PIA/VIA spectrometer used for the steady-state hole concentration experiments.

A MOSFET circuit between the 365 nm pump LED and its power supply is connected to the output of the DAQ card, allowing software-controlled switching on/off of a 365 nm LED. The 365 nm LED is incident on the rear (FTO side) of the sample. A PalmSens3 potentiostat is used to hold a constant applied bias for the PIA experiments (or the stepped applied bias for VIA) and records the resulting current response. These experiments follow the hole concentration under steady-state conditions, so the PIA/VIA amplitude is taken after it has reached an equilibrium value following the photo/electrochemical perturbation (averaged over the final few seconds of the plateau regions in Figure S16a & S16c).

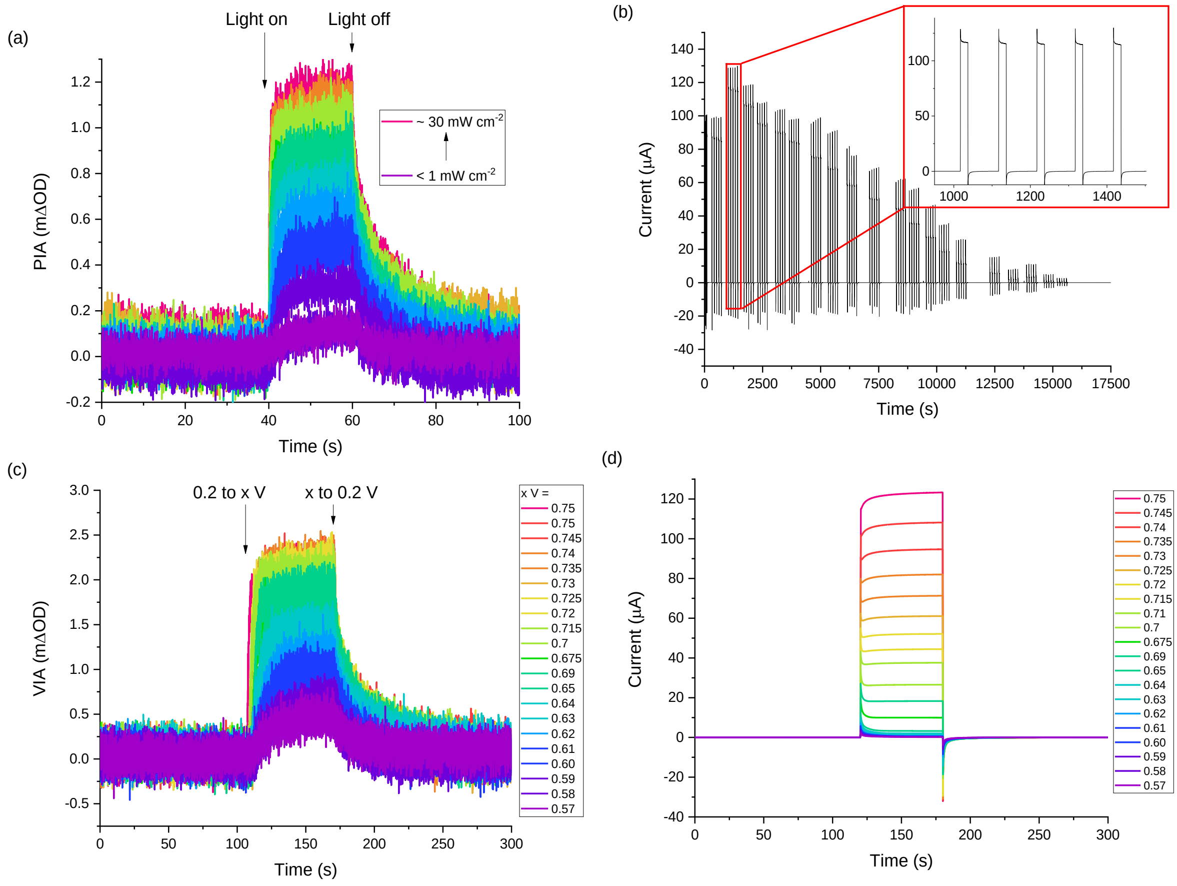

Figure S16: Average PIA amplitude probed at 625 nm over 5 light on/off cycles (a) and photocurrents (b) for an α-Fe_2O_3 film in 0.1 M NaOH held at 0.2 V vs. Ag/Ag^+ at various 365 nm pump LED light intensities. VIA amplitude probed at 625 nm (c) and simultaneous current response (d) for the same film over approximately the same range of current densities, stepping from 0.2 V vs Ag/Ag^+ to the stated values. Both experiments were allowed to reach a plateau in ΔOD values, which took around 20 seconds for PIA and 60 seconds for VIA experiments. PIA and photocurrent values were averaged over 5 light on/off cycles to achieve comparable signal-to-noise with the VIA experiments.

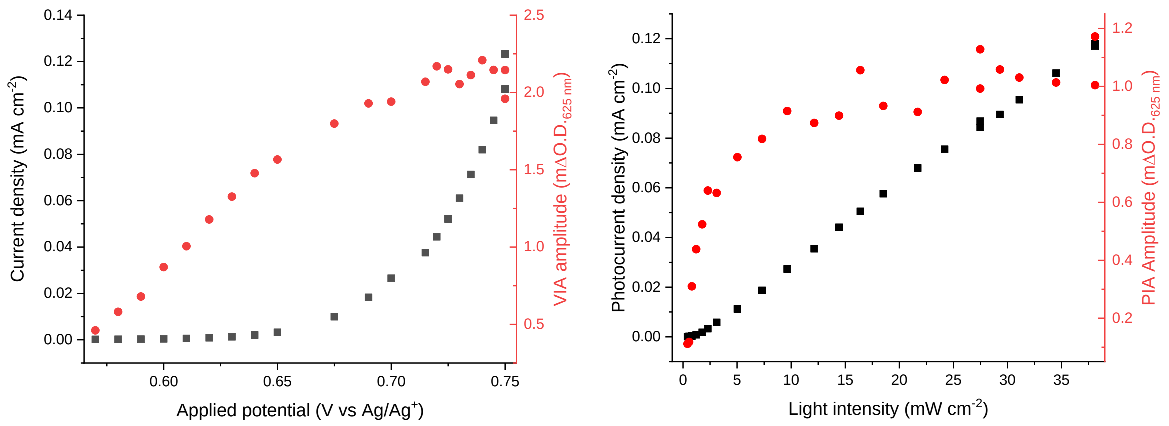

Figure S17: VIA (left) and PIA (right) responses of the electrode obtained from the raw data above.

These steady-state absorption and photocurrent measurements are then used to estimate the reaction order (α) and relative rate constant for water oxidation (k_{wo}) using the kinetic analysis described by Le Formal *et al.*[16] The change in absorbance at 625 nm is directly proportional to the surface hole concentration, while the simultaneous current density directly reports on the rate of charge consumption (*i.e.* the flux of holes to the surface, J_{h+}). Under steady-state conditions when the PIA amplitude reaches a plateau the surface hole concentration does not experience any net change:

$$\frac{d[h^+]_s}{dt} = 0 \qquad \text{Eq. S7}$$

The water oxidation current also reaches a plateau in this region, indicating the flux of holes to the surface is equal to the rate of water oxidation by surface holes:

$$\frac{d[h^+]_s}{dt} = J_{h^+} - k_{wo} \times [h^+]_s^{\alpha} \qquad \text{Eq. S8}$$

Combining Equations S5 & S6 and linearising yields:

$$J_{holes} = k_{wo} \times [h^+]_s{}^{\alpha} \qquad \text{Eq. S9}$$

$$log_{10}(J_{holes}) = log_{10}(k_{wo}) + \alpha \times log_{10}[h^+]_s \qquad \text{Eq. S10}$$

Which indicates the gradient of a log-log plot of PIA amplitude versus current density is proportional to α. Calculation of k_{wo} from Equation S9 using α = 1 and α = 3 shows that the relative rate constant for water oxidation is constant within the 1st and 3rd order regimes. We also plot the highest current density data for α = 8 which again shows that the relative rate constant for water oxidation is roughly constant with hole density but we caution that there are significant errors in the fitting of the VIA data under these conditions.

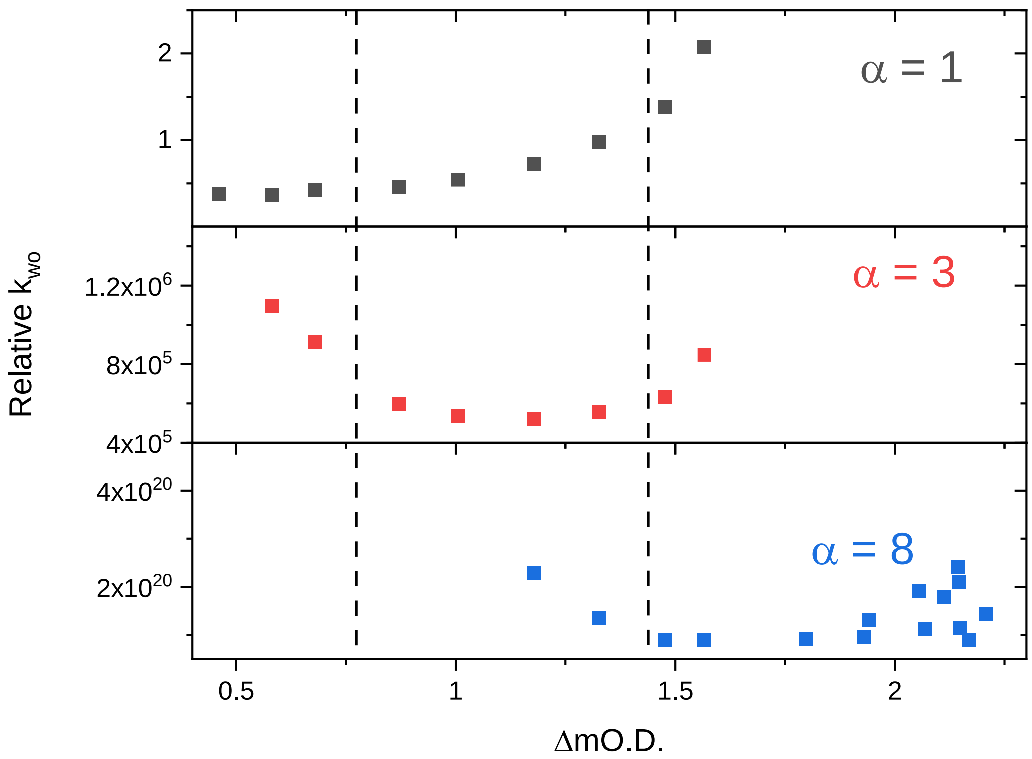

Figure S18: Relative water oxidation rate constant (k_{wo}) for α-Fe_2O_3 in 0.1 M NaOH obtained by use of $J = k_{WO} \cdot [h^+]^\alpha$ for α = 1 (grey top panel) and α = 3 (red lower panel) where the relative hole concentration is the change in optical density (ΔO.D.) with applied potential (vs. a reference potential (0.2 V)) and *J* is the oxygen evolution current density, measured in the dark. At low $[h^+]$ (low ΔO.D.) to the left of the dashed line k_{wo} is constant for α = 1. In the middle k_{wo} is constant for α = 3 on the right-hand side using α = 8 we also see that the k_{wo} is not strongly dependent on hole density.

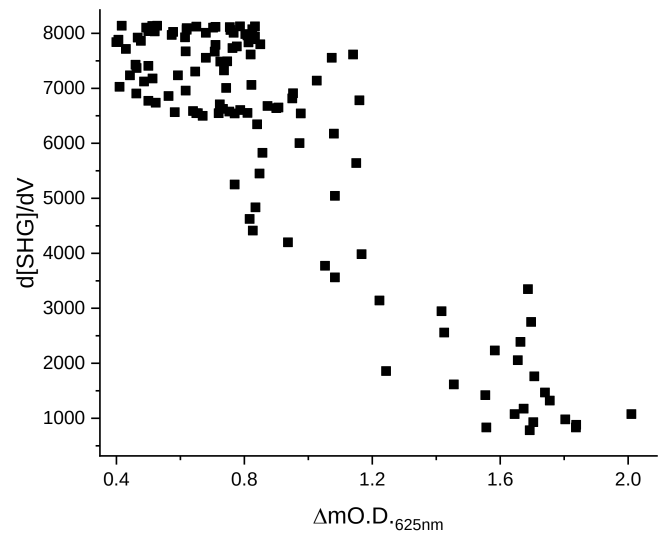

Figure S19: Plot of the derivative of SHG peak area vs change in optical density at 625 nm (*i.e.* proportional to surface hole concentration) from the VIA measurements.

8. References

1 O. Zandi, A. R. Schon, H. Hajibabaei and T. W. Hamann, *Chem. Mater.*, 2016, **28**, 765–771.
2 L. A. Marusak, R. Messier and W. B. White, *J. Phys. Chem. Solids*, 1980, **41**, 981–984.
3 C. Li, D. Wang, N. Suzuki, C. Terashima, Y. Liu, A. Fujishima and X. Zhang, *Electrochimica Acta*, 2020, **360**, 137012.
4 A. Kay, I. Cesar and M. Grätzel, *J. Am. Chem. Soc.*, 2006, **128**, 15714–15721.
5 S. V. Makarov, M. I. Petrov, U. Zywietz, V. Milichko, D. Zuev, N. Lopanitsyna, A. Kuksin, I. Mukhin, G. Zograf, E. Ubyivovk, D. A. Smirnova, S. Starikov, B. N. Chichkov and Y. S. Kivshar, *Nano Lett.*, 2017, **17**, 3047–3053.
6 S. R. Pendlebury, X. Wang, F. L. Formal, M. Cornuz, A. Kafizas, S. D. Tilley, M. Grätzel and J. R. Durrant, *J. Am. Chem. Soc.*, 2014, **136**, 9854–9857.
7 C. P. Marshall, W. J. B. Dufresne and C. J. Rufledt, *J. Raman Spectrosc.*, 2020, **51**, 1522–1529.
8 A. Hankin, F. E. Bedoya-Lora, J. C. Alexander, A. Regoutz and G. H. Kelsall, *J. Mater. Chem. A*, 2019, **7**, 26162–26176.
9 R. A. Lunt, A. J. Jackson and A. Walsh, *Chem. Phys. Lett.*, 2013, **586**, 67–69.
10 P. Guyot-Sionnest and A. Tadjeddine, *J. Chem. Phys.*, 1990, **92**, 734–738.
11 N. G. Rey and D. D. Dlott, *J. Electroanal. Chem.*, 2017, **800**, 114–125.
12 S. Ong, X. Zhao and K. B. Eisenthal, *Chem. Phys. Lett.*, 1992, **191**, 327–335.
13 J. M. Lantz, R. Baba and R. M. Corn, *J. Phys. Chem.*, 1993, **97**, 7392–7395.
14 L. M. Peter, in *Photocatalysis: Fundamentals and Perspectives*, RSC, 2016, pp. 1–28.
15 M. Schmid, D. Rath and U. Diebold, *ACS Meas. Sci. Au*, 2022, **2**, 185–196.
16 F. L. Formal, E. Pastor, S. D. Tilley, C. A. Mesa, S. R. Pendlebury, M. Grätzel and J. R. Durrant, *J. Am. Chem. Soc.*, 2015, **137**, 6629–6637.
